# CircRHBDD1 promotes immune escape via IGF2BP2/PD-L1 signaling and acts as a nanotherapeutic target in gastric cancer

**DOI:** 10.1186/s12967-024-05498-9

**Published:** 2024-07-30

**Authors:** Yanna Li, Zhixiong Wang, Peng Gao, Danping Cao, Runyu Dong, Menglin Zhu, Yao Fei, Xueliang Zuo, Juan Cai

**Affiliations:** 1 Department of Gastrointestinal Surgery, The First Affiliated Hospital, Yijishan Hospital of Wannan Medical College, Wuhu, 241001 China; 2 Anhui Province Key Laboratory of Non-coding RNA Basic and Clinical Transformation, Wannan Medical College, Wuhu, 241001 China; 3 Department of Oncology, The First Affiliated Hospital, Yijishan Hospital of Wannan Medical College, Wuhu, 241001 China

**Keywords:** Gastric cancer, Immune escape, PD-L1, Ubiquitination, N6-methyladenosine, CircRNAs, Nanotherapy

## Abstract

**Background:**

Circular RNAs (circRNAs) have been implicated in the development and progression of gastric cancer (GC). However, it remains unclear whether dysregulated circRNA affects immune escape and the efficacy of immunotherapy in GC. Our aim is to investigate the molecular mechanism of circRNA affecting GC immunotherapy and identify effective molecular therapeutic targets.

**Methods:**

The differential expression profile of circRNAs was established through circRNA sequencing, comparing three paired GC tissues with their adjacent non-cancerous gastric tissues. The expression level of circRHBDD1 in GC tissues was then assessed using quantitative reverse transcription polymerase chain reaction (qRT-PCR). The biological characteristics of circRHBDD1 were verified through a series of experiments, including agarose gel electrophoresis assays, RNase R treatment, and actinomycin D experiments. The prognostic value of circRHBDD1 in GC was evaluated by conducting both univariate and multivariate survival analyses. Furthermore, loss- and gain-of-function approaches were utilized to investigate the impact of circRHBDD1 on GC immune escape. RNA-sequencing, immunoprecipitation, flow cytometry, and methylated RNA immunoprecipitation (meRIP) analysis were performed to elucidate the underlying molecular mechanisms.

**Results:**

We discovered that circRHBDD1 exhibited remarkably high expression levels in GC tissues and cell lines. Notably, the high expression of circRHBDD1 was significantly correlated with poor overall survival and disease-free survival among GC patients. Both in vitro and in vivo experiments revealed that circRHBDD1 upregulated the expression of PD-L1 and impeded the infiltration of CD8^+^ T cells. Further, we found that circRHBDD1 binds to IGF2BP2, disrupting the interaction between E3 ligase TRIM25 and IGF2BP2, and ultimately inhibiting IGF2BP2 ubiquitination and degradation. Intriguingly, IGF2BP2 enhances PD-L1 mRNA stability through m^6^A modification. Additionally, we developed Poly (lactide-co-glycolic acid) (PLGA)-Polyethylene glycol (PEG)-based nanoparticles loaded with circRHBDD1 siRNA. In vivo experiments validated that the combination of PLGA-PEG(si-circRHBDD1) and anti-PD-1 offers a safe and efficacious nano-drug regimen for cancer immunotherapy.

**Conclusion:**

Our results demonstrated that circRHBDD1 promoted GC immune escape by upregulating the expression of PD-L1 and reprogramming T cell-mediated immune response. Inhibition of circRHBDD1 expression could potentially enhance the response of GC patients to immunotherapy, thus improving treatment outcomes. Additionally, the development of a nanodrug delivery system provides a feasible approach for future clinical applications.

**Supplementary Information:**

The online version contains supplementary material available at 10.1186/s12967-024-05498-9.

## Introduction

According to the latest GLOBOCAN report, gastric cancer (GC) is the fifth most common cancer and the fourth leading cause of cancer-related death globally [1]. Pathologically, more than 95% of GC is adenocarcinoma [2]. The mortality rate of male patients with GC is twice than that of female patients [3]. The incidence and mortality of GC patients in the world have decreased, but the incidence in East Asian countries is still very high, accounting for more than 70% of the global cases [4]. Japan has the highest prevalence of gastrointestinal cancer, while Mongolia has the highest mortality rate [5]. Although the treatment modalities for GC have made great progress, more than half of the patients have reached the intermediate and advanced stages when presenting clinical manifestations [6]. Clinical efficacy of traditional therapies such as systemic chemotherapy and radiotherapy is limited, and the 5-year survival rate for patients with advanced GC is only 20-30% [7]. In the past few years, the GC combined immunotherapy has undergone rapid development, providing different immunotherapy or targeted therapy for patients with GC at advanced stages. For example, anti-PD-1 and anti-HER2 agents have achieved surprising therapeutic effects in GC [8]. Therefore, it is of great value to explore the molecular mechanism underlying GC progression and determine effective molecular therapeutic targets.

Circular RNAs (circRNAs), a class of non-coding RNAs generated by back-splicing of pre-mRNAs, have a covalently closed-loop structure, which makes them more stable than linear RNAs [9]. The growth of RNA sequencing and bioinformatics has made it possible to determine the widespread distribution of circRNAs in various cell types and tissues. and biological fluids [10]. CircRNAs play a role in physiological and pathological processes, especially in tumor occurrence and progression [11]. They function through multiple mechanisms, including interacting with microRNAs and proteins [12, 13]. For example, circRHOT1 aggravates hepatocellular carcinoma progression by recruiting histone acetyltransferase KAT5 (TIP60) to NR2F6, while circ-Foxo3 promotes the occurrence of breast cancer by acting as a protein scaffold and binging to E3 ubiquitin-protein ligase MDM2 and p53 [14]. Nonetheless, circRNAs’ function in controlling anti-tumor immunity in GC remains unclear.

Numerous investigations have revealed that circRNAs are crucial for tumor immunity. CircUBAP2 could regulate the expression of CXCR4 and ZEB1, inhibit antigen presentation and promote immune escape by regulating the infiltration and function of immune cells in pancreatic cancer [15]. CircRNAs can promote tumor immune escape and tumor proliferation [16]. Blocking of PD-1/PD-L1 signaling has brought new hope for GC patients [17]. However, many patients are resistant to immune checkpoint inhibitors, and the mechanisms include the lack of suitable tumor antigens, dysfunction of Major Histocompatibility Complex on the surface of tumors, abnormal IFN-γ signaling pathway, and immunosuppressive tumor microenvironment [18]. It is essential to study the molecular regulatory mechanism of PD-L1 to improve the efficacy of anti-PD-1/PD-L1 therapy in GC.

Nanoparticles (NPs) offer several advantages in cancer therapy, including enhanced permeability and retention effect, targeted delivery, and reduced systemic toxicity [19, 20]. In addition, NPs prevent the degradation of siRNAs, prolong the drug circulation time and passive targeting capability [21]. PLGA NPs have been serve as carriers for drug delivery [22]. PLGA-NPs, which exhibit excellent biocompatibility and biodegradability, have been approved by the Food and Drug Administration (FDA) for clinical application. For example, PD-1-MM@PLGA/RAPA, a novel nanoplatform, can cross the blood-brain barrier offering a new approach for the treatment of glioblastoma [23]. PLGA-PEG NPs have been used for controlled drug release and are promising for cancer treatment [24, 25]. NPs may also facilitate T cell activation and improve anti-tumor efficacy with minimal toxicity [26].

In this study, we found circRHBDD1 by RNA sequencing and it was derived from a back-splicing event between exons 6 and 8 of RHBDD1. CircRHBDD1 was markedly overexpressing in GC. CircRHBDD1 can upregulate the expression of PD-L1 in GC and inhibit the infiltration of CD8^+^ T cells. In vivo experiments showed that circRHBDD1 has no influence on tumor growth in immunodeficient mice. However, overexpression of circRHBDD1 in C57BL/6 mouse model could promote tumor growth. Mechanistically, circRHBDD1 could bind with IGF2BP2 and impeding the interaction between TRIM25 and IGF2BP2, thus inhibiting the ubiquitination of IGF2BP2. IGF2BP2 can promote the stability of PD-L1 mRNA via m^6^A modification. Moreover, targeting circRHBDD1 facilitated the efficacy of anti-PD-1 treatment in GC. This study revealed that circRHBDD1 could promote immune escape of GC through the IGF2BP2/PD-L1 axis and be a nanotherapeutic candidate.

## Methods

### Patients and tissue samples

All GC tissues and adjacent nontumorous tissues were collected from patients who underwent gastrectomy in the Department of Gastrointestinal Surgery at the First Affiliated Hospital of Wannan Medical College. The patients did not receive any anti-tumor therapy before surgery and were diagnosed with GC by histopathological assessment. Tissue samples were immediately snap-frozen in liquid nitrogen and stored at -80 °C until further use. The study was approved by the Ethics Committee of the First Affiliated Hospital of Wannan Medical College (Approval Number: 2021-45). Informed consent was obtained from all patients prior to sample collection.

### PD-L1 and PD-1 binding assay

The measurements for the PD-L1 and PD-1 binding test were made using the previously mentioned protocols. [27]. Cells were seeded into confocal dish at a density of 3 × 10^4^ cells/dish. After 24 h, cells were fixed with 4% paraformaldehyde for 15 min, followed by treatment with recombinant human PD-1 Fc protein (R&D Systems, USA) for 1 h at room temperature. Subsequently, cells were incubated with anti-human Alexa Fluor 488 dye (Invitrogen, USA) for 1 h. Nuclei were stained with DAPI for 5 min. Fluorescence intensity of Alexa Fluor 488 was measured using a Synergy Neo microplate reader (BioTeK, VT, USA) and normalized to total protein content. Confocal laser-scanning microscopy (Carl Zeiss) was used to visualize the cells.

### Co-IP (Co-immunoprecipitation) assay

For Co-IP assays, cells were collected and washed three times with PBS. Total protein was extracted using RIPA lysis buffer supplemented with PMSF. The lysates were incubated with primary antibodies anti-IGF2BP2(CST, 1:100) and anti-TRIM25 (abcam, 1:100) at room temperature for 2 h. IgG of the same species as the endogenous antibody was used as a negative control. Followed by overnight incubation with protein A/G PLUS-Agarose beads (Santa Cruz Biotechnology) at 4 °C. Beads were washed three times with PBS, resuspended in SDS-PAGE loading buffer, and subjected to Western blot analysis. For sample loading, whole cell lysates were used as Input group, the positive control group.

### Preparation and characterization of PLGA-PEG(si-circRHBDD1) NPs

PLGA-PEG(si-circRHBDD1) nanoparticles were prepared using a double emulsion solvent diffusion method [28]. Briefly, 1 mg of si-circRHBDD1 was dissolved in 4 ml of DEPC water and emulsified in 0.5 ml chloroform by sonication (30 s, 100 W) over an ice bath. This primary emulsion was further emulsified in 4 ml of 2.5% (w/v) PVA solution using sonication (2 min, 80 W) over an ice bath to form a water-in-oil-in-water emulsion. The organic solvent was evaporated by stirring at room temperature for 3 h. The NPs were collected by centrifugation at 10,000 rpm for 15 min and washed twice with DEPC water. TEM (JEOL JEM-1230) was used to examine NP morphology. Size, zeta potential, and PDI were measured using a Zetasizer Nano ZSE (Malvern Instruments Ltd., UK).

The morphology and size of NPs were examined by Transmission electron microscope (TEM). The size, zeta potential and polydispersity index (PDI) were observed by Dynamic light scattering (DLS) with a Nano Particle Analyzer (Zetasizer Nano ZSE, Malvern Instruments Ltd., UK).

### Lysosome escape experiments

MKN-28 cells were seeded onto 15-mm confocal plates at a density of 5 × 10^4^cells per well. After 24 h, cells were incubated with Coumarin-6 NPs (1.5 µg/mL) for 2 h. Cells were then stained with LysoTracker Red (75 nM) and DAPI (1 µg/mL) for 20 min. Following a final PBS wash, fluorescence was visualized using a confocal laser-scanning microscope (Carl Zeiss) with excitation/emission settings of 488/525 nm for Coumarin-6 and 561/610 nm for LysoTracker Red.

### In vivo anti-tumor efficacy and toxicity evaluation of NPs

C57BL/6 mice (6–8 weeks old, female) were used to create tumor models by subcutaneous injection of GC cells. When tumors reached ~ 100 mm^3^, mice were injected with PLGA-PEG(si-circRHBDD1) NPs or PLGA-PEG(siRNA control) NPs (200 mg/kg) via tail vein. Anti-PD-1 (100 µg per mouse) or control IgG isotype was administered intraperitoneally on days 3, 6, 9, and 12. Tumor volume was measured with calipers and calculated as V = L × W^2^/2. Tumors and major organs (liver, kidney, lung, spleen, heart) were excised, weighed, and fixed with 4% paraformaldehyde for H&E staining. Dynamic fluorescence imaging was performed using an in vivo imaging system (Biolight Biotechnology Co, Ltd., Guangzhou, China) 48 h post-injection of DiR-labeled NPs.

### Statistical analysis

Statistical analyses were performed using GraphPad Prism 8 (San Diego, CA, USA) and SPSS 26.0 (Chicago, IL, USA). Protein fluorescence intensity was quantified using ImageJ software (NIH, USA). Data are presented as mean ± SD. One-way ANOVA was used for comparisons among multiple groups, while the Student’s t-test was used for two-group comparisons. Kaplan-Meier survival curves were generated, and multivariate survival analysis was conducted using the Cox proportional hazards model. A p-value of < 0.05 was considered statistically significant.

## Results

### CircRHBDD1 was a highly expressed circRNA in GC and is associated with poor prognosis

**Fig. 1:**
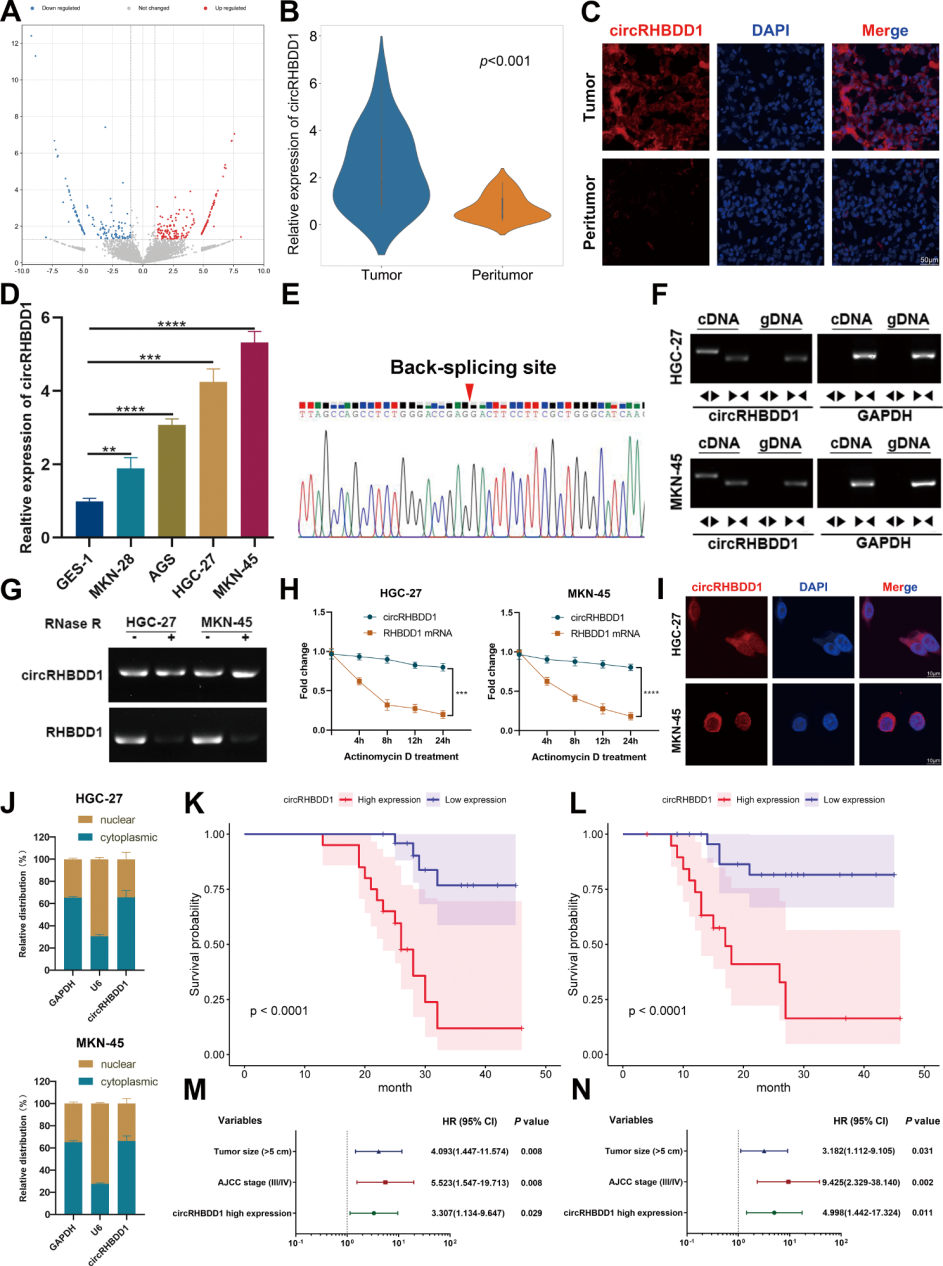
CircRHBDD1 was a circRNA highly expressed in GC and predicted poor prognosis. **A** Volcano map of the discrepantly expressive circRNAs in GC. **B** qRT-PCR was utilized to test CircRHBDD1 expression level in 45 pairs of GC tissues and adjacent tissues. **C** FISH was utilized to examined circRHBDD1 expression level in GC and adjacent tissues. Scale bar = 50 μm. **D** CircRHBDD1 expression level in four GC cell strains and normal gastric mucosa cell strains was measured via qRT-PCR. **E** Sanger sequencing showed the reverse splicing locus of circRHBDD1. **F** Gelose Gel Electropho to verify the stability of circRHBDD1. **G** RNase R assay was used to analyze the consistency of circRHBDD1 and RHBDD1 in HGC-27 and MKN-45 cells. **H** After the HGC-27 and MKN-45 cells were managed with actinomycin D at specific time points, qRT-PCR was utilized to test the mRNA expression levels of circRHBDD1 and RHBDD. **I** The localization of circRHBDD1 in HGC-27 and MKN-45 cells was tested by FISH. Scale bar = 10 μm. **J** The localization of circRHBDD1 was examined by nuclear and cytoplasmic fractions assay. **K** The pertinence of expression levels between circRHBDD1 and OS (*p* ˂ 0.0001). **L** The pertinence of expression levels between circRHBDD1 and DFS (*p* ˂ 0.0001). **M** Multivariate analysis revealed that the independent risk factors for OS. **N** Multivariate analysis showed that the independent risk factors affecting DFS. Data are presented with the means ± SD of three independent experiments. **p < 0.01; ***p < 0.001; ****p < 0.0001

CircRNA sequencing of three pairs of GC tissues and adjacent non-tumorous tissues revealed differentially expressed circRNAs (Fig. 1A). qRT-PCR analysis confirmed that circRHBDD1 was significantly upregulated in GC tissues compared to adjacent non-tumorous tissues (Fig. 1B, *p* < 0.001). FISH analysis corroborated the overexpression of circRHBDD1 in GC tissues (Fig. 1C). qRT-PCR confirmed that the expression of circRHBDD1 in gastric cancer cells was higher than that in normal gastric mucosa cells. (Fig. 1D). Sanger sequencing verified the back-splicing junction of circRHBDD1 (Fig. 1E), and electrophoresis showed its amplification in cDNA but not gDNA (Fig. 1F). RNase R and Actinomycin D assays demonstrated the stability of circRHBDD1 compared to linear RHBDD1 mRNA (Fig. 1G, H). FISH and RNA fractionation assays indicated that circRHBDD1 is predominantly localized in the cytoplasm (Fig. 1I, J). High circRHBDD1 expression was significantly associated with poor differentiation (*p* = 0.002) and larger tumor size (*p* = 0.015) (Table S1). Kaplan-Meier survival analysis showed that high circRHBDD1 expression correlated with shorter overall survival (OS) and disease-free survival (DFS) (Fig. 1K, L, *p* < 0.0001). Cox regression analysis identified high circRHBDD1 expression as an independent prognostic factor for OS and DFS (Fig. 1M, N; Tables S2, S3). These findings suggested that circRHBDD1 functioned as a highly expressed circRNA in GC, and high expression of circRHBDD1 predicted poor prognosis in GC patients.

### CircRHBDD1 had no effects on the proliferation of GC in vitro and in nude mouse models

To investigate the role of circRHBDD1 in GC cell proliferation, we performed knockdown and overexpression experiments. qRT-PCR confirmed efficient knockdown by sh-circRHBDD1-1 and sh-circRHBDD1-2 (Fig. S1A). CCK-8, EdU, and colony formation assays showed no significant effect of circRHBDD1 knockdown on GC cell proliferation (Fig. S1B-D). Similarly, circRHBDD1 overexpression did not affect proliferation (Fig. S2A-D). In vivo, circRHBDD1-silenced HGC-27 cells and circRHBDD1-overexpressing MKN-28 cells were injected into nude mice, but no significant differences in tumor volume, weight, or survival were observed (Fig. S3A-C). These results suggest that circRHBDD1 does not affect GC cell proliferation directly, implying an immune-mediated mechanism.

### CircRHBDD1 upregulated the expression of PD-L1 in GC cells

**Fig. 2:**
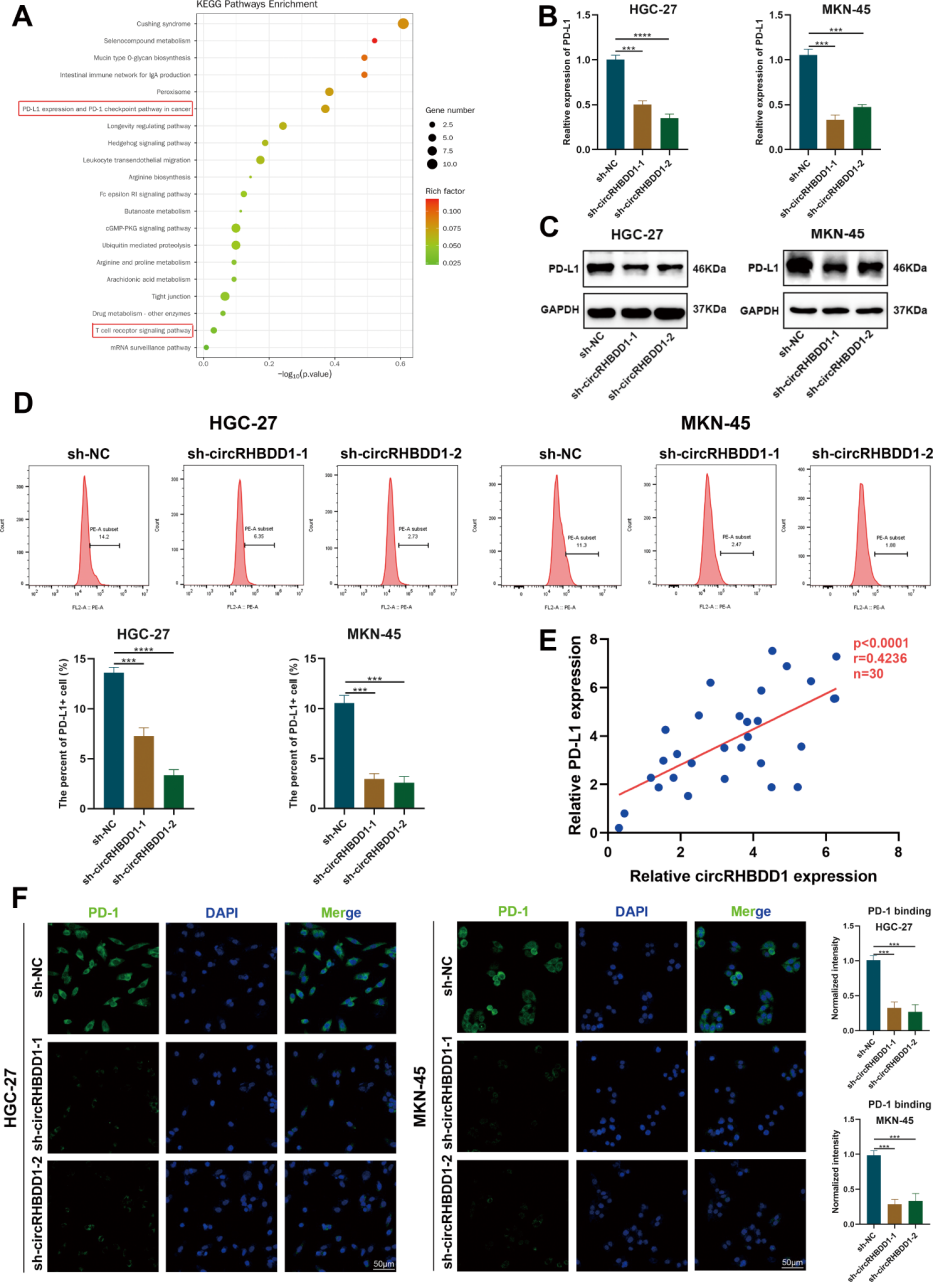
CircRHBDD1 upregulated the expression of PD-L1 in GC cells. **A** KEGG analysis indicated enriched pathways. **B**, **C** qRT-PCR and Western blotting were carried out to detected the expression levels of PD-L1 mRNA and protein after circRHBDD1 knockdown in HGC-27 and MKN-45 cells. **D** Flow cytometry was utilized to examined the surface PD-L1 expression of the cytomembrane after circRHBDD1 knockdown in HGC-27 and MKN-45 cells. **E** Correlation analysis between circRHBDD1 and the expression level of PD-L1 in GC tissues (*n* = 30). **F** Immunofluorescence assays to detect the combination intensity between PD-L1 and PD-1 on HGC-27 and MKN-45 cells conducted by shRNA for circRHBDD1. Scale bar = 50 μm. Data are appeared with the means ± SD of three independent experiments. ***p < 0.001; ****p < 0.0001

RNA sequencing and KEGG pathway analysis identified the PD-L1/PD-1 checkpoint pathway as significantly affected by circRHBDD1 knockdown (Fig. 2A). qRT-PCR and Western blot confirmed that circRHBDD1 knockdown reduced PD-L1 expression, while overexpression increased it (Fig. 2B, C; Fig. S4A, B). Flow cytometry showed that circRHBDD1 knockdown decreased, and overexpression increased, PD-L1 membrane expression (Fig. 2D; Fig. S4C). In addition, correlation analysis suggested that the expression level of circRHBDD1 was directly associated with PD-L1 expression level (Fig. 2E). It is recognized that PD-L1 regulates T-cell tolerance by interacting with its receptor PD-1 [29]. Immunofluorescence indicated that circRHBDD1 affects the PD-1/PD-L1 interaction on GC cells (Fig. 2F; Fig. S4D). These results demonstrate that circRHBDD1 upregulates PD-L1 expression, contributing to immune escape in GC.

### Knockdown of circRHBDD1 facilitated the infiltration and killing ability of CD8^+^ T cells

**Fig. 3:**
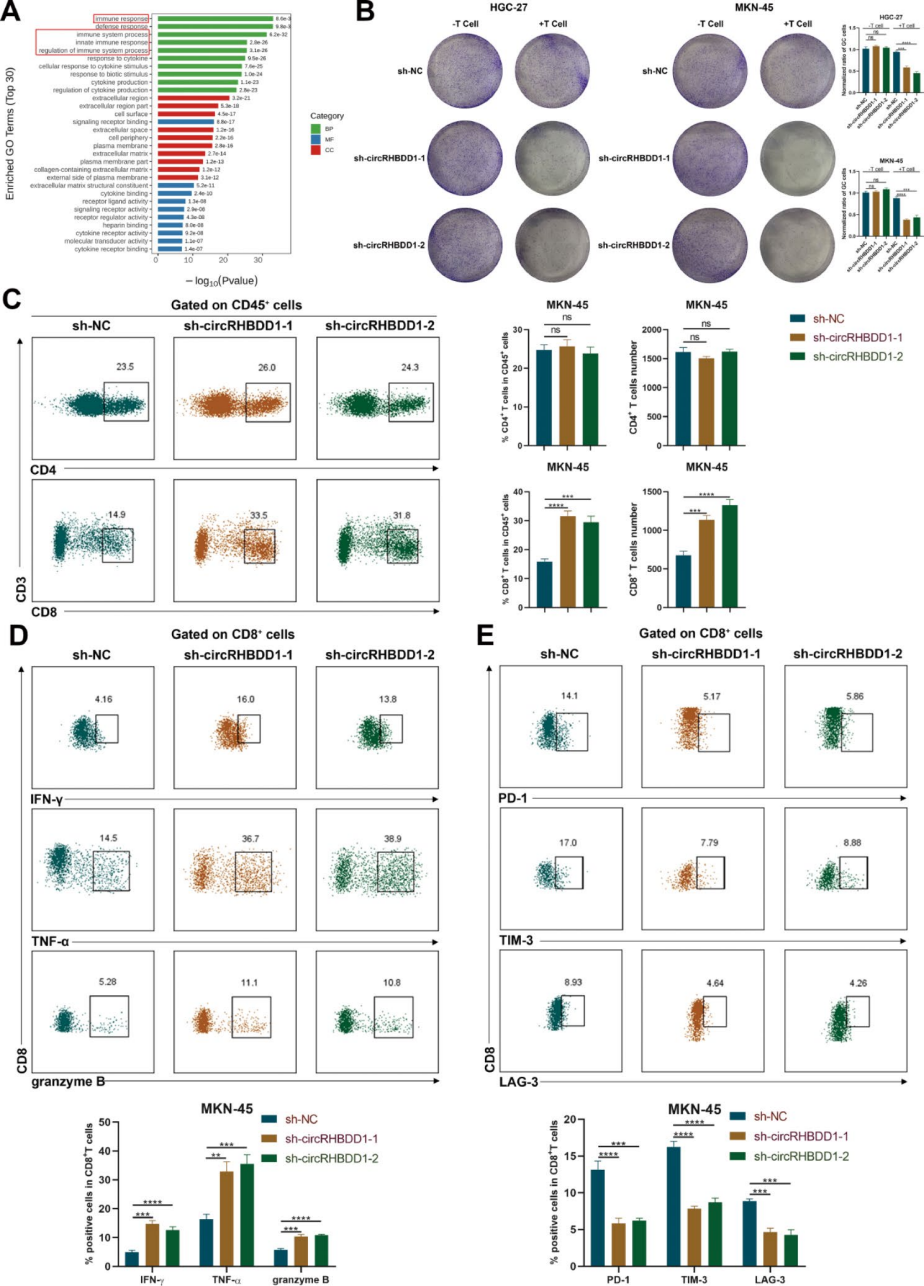
Knockdown of circRHBDD1 accelerated the infiltration and killing ability of CD8^+^ T cells. **A** GO analysis showed the enriched pathways. **B** The consequences of T cell-induced tumor cell cytotoxicity assays in circRHBDD1-silenced HGC-27 and MKN-45 cells. **C** The content and percentum of CD4^+^ T cells and CD8^+^ T cells in CD45^+^ cells were examined by flow cytometry after sh-circRHBDD1-1 and sh-circRHBDD1-2 infection. **D** The expressions of IFN-γ, TNF-α and granzyme B were measured by flow cytometry after circRHBDD1 knockdown. **E** The expressions of PD-1, TIM-3 and LAG-3 in CD8^+^ T cells were examined by flow cytometry in MKN-45 cells with circRHBDD1 knockdown. Data are presented with the means ± SD of three independent experiments. **p < 0.01; ***p < 0.001; ****p < 0.0001; ns not significant

GO analysis revealed significant enrichment of immune response-related genes (Fig. 3A). Given the significant role of T cells in anti-tumor immunity [30], we conducted a T cell-mediated tumor cell killing assay by co-culturing transfected gastric cancer cells with isolated and activated human PBMCs. Co-culture assays with human PBMCs showed reduced GC cell numbers in the circRHBDD1 knockdown group (Fig. 3B), and increased in the overexpression group (Fig. S5A). Flow cytometry indicated higher percentages and counts of CD8^+^ T cells, but not CD4^+^ T cells, in the knockdown group (Fig. 3C). Knockdown also increased IFN-γ, TNF-α, and granzyme B secretion by CD8^+^ T cells, and decreased PD-1, TIM-3, and LAG-3 expression (Fig. 3D, E). Overexpression of circRHBDD1 had the opposite effect (Fig. S5B-D). These findings suggest that circRHBDD1 knockdown enhances CD8^+^ T cell infiltration and activity, exerting an anti-tumor effect.

### CircRHBDD1 interacted with IGF2BP2 and inhibited its ubiquitination and degradation

**Fig. 4:**
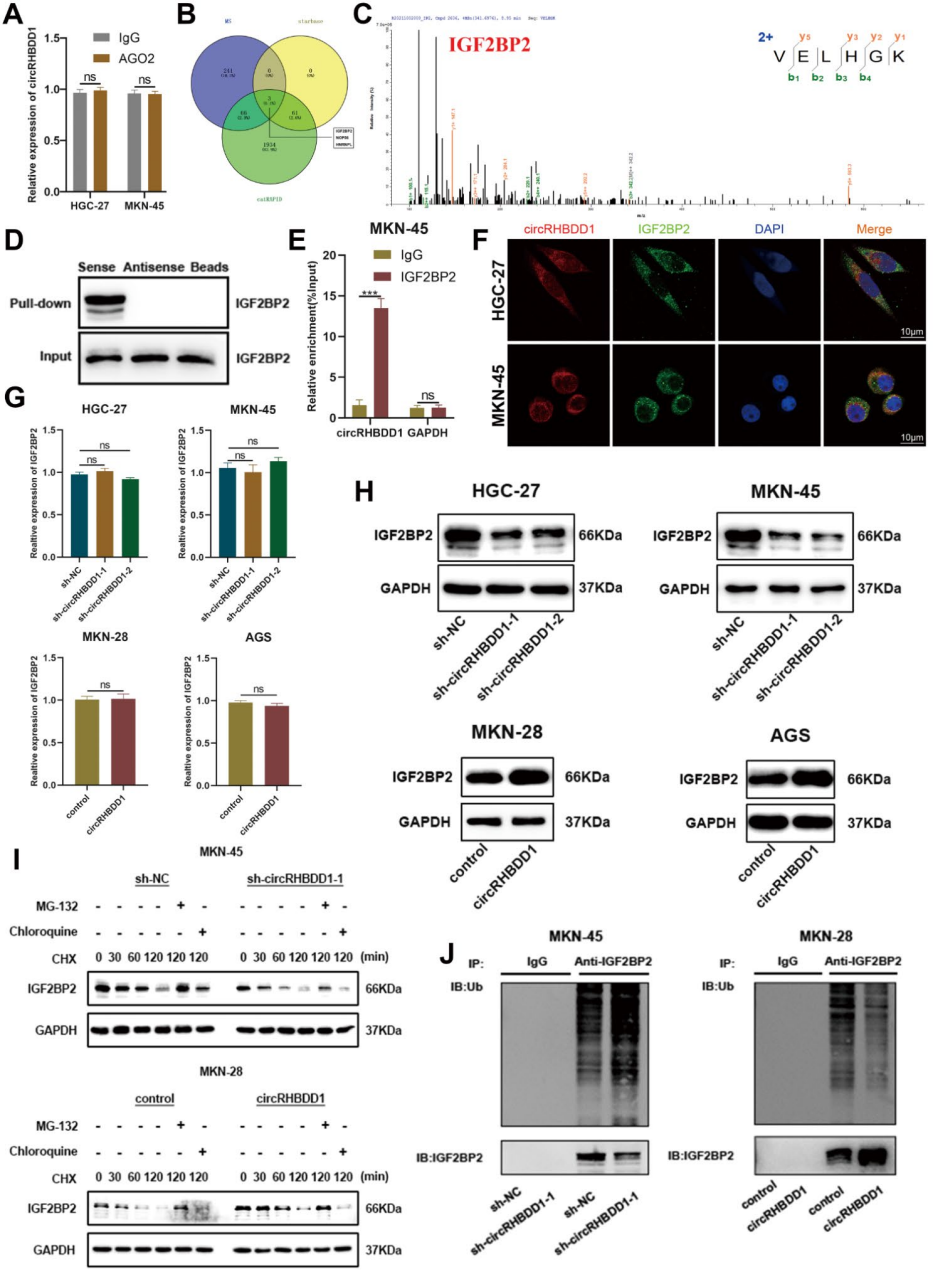
CircRHBDD1 interacted with IGF2BP2 and inhibited its ubiquitination and degradation. **A** The enrichment status of circRHBDD1 in the AGO2 IP was detected using qRT-PCR through the RIP assay. **B** Venn diagram showing the intersection of starbase and catRAPID database prediction and mass spectrometry analysis. **C** IGF2BP2 protein bound to circRHBDD1, as determined by mass spectrometry. **D** Detection of proteins by Western blotting after RNA pull-down. **E** RIP detections indicated the combination between IGF2BP2 and circRHBDD1. **F** FISH of circRHBDD1 and immunofluorescence detection of IGF2BP2 in HGC-27 and MKN-45 cells. Scale bar = 10 μm. **G** qRT-PCR was utilized to detect the mRNA expression level of IGF2BP2 after knocking down or overexpressing circRHBDD1. **H** Western blotting was utilized to analyze the expression level of IGF2BP2 protein after knocking down or overexpressing circRHBDD1. **I** In circRHBDD1-silenced MKN-45 and MKN-28 cells, Western blotting revealed the protein levels of IGF2BP2 under treatment with MG-132 or chloroquine. **J** After treated with MG-132, western blotting was employed to assess the role of circRHBDD1 on the ubiquitination level of IGF2BP2 in MKN-45 and MKN-28 cells. Data are presented with the means ± SD of three independent experiments. ***p < 0.001; ns not significant

AGO2-RIP assays suggested that circRHBDD1 does not act as a miRNA sponge (Fig. 4A). Starbase (https://starbase.sysu.edu.cn/) [31] and catRAPID (http://service.tartaglialab.com/page/catrapid_group) [32] was employed to predict the RBPs which could combine with circRHBDD1. RNA binding protein (RBP) prediction and mass spectrometry identified IGF2BP2, NOP56, and HNRNPL as potential binding partners (Fig. 4B, C). Western blotting and RIP confirmed the interaction between circRHBDD1 and IGF2BP2 (Fig. 4D, E). RNA FISH showed colocalization in the cytoplasm (Fig. 4F). CircRHBDD1 knockdown decreased, while overexpression increased, IGF2BP2 protein levels without affecting mRNA levels (Fig. 4G, H). Proteasome inhibition assays indicated that circRHBDD1 inhibits IGF2BP2 degradation via the ubiquitin-proteasome pathway (Fig. 4I). Ubiquitination assays confirmed that circRHBDD1 inhibits IGF2BP2 ubiquitination (Fig. 4J).

### CircRHBDD1 inhibited the ubiquitination of IGF2BP2 by impeding the interaction of E3 ligase TRIM25 and IGF2BP2

**Fig. 5:**
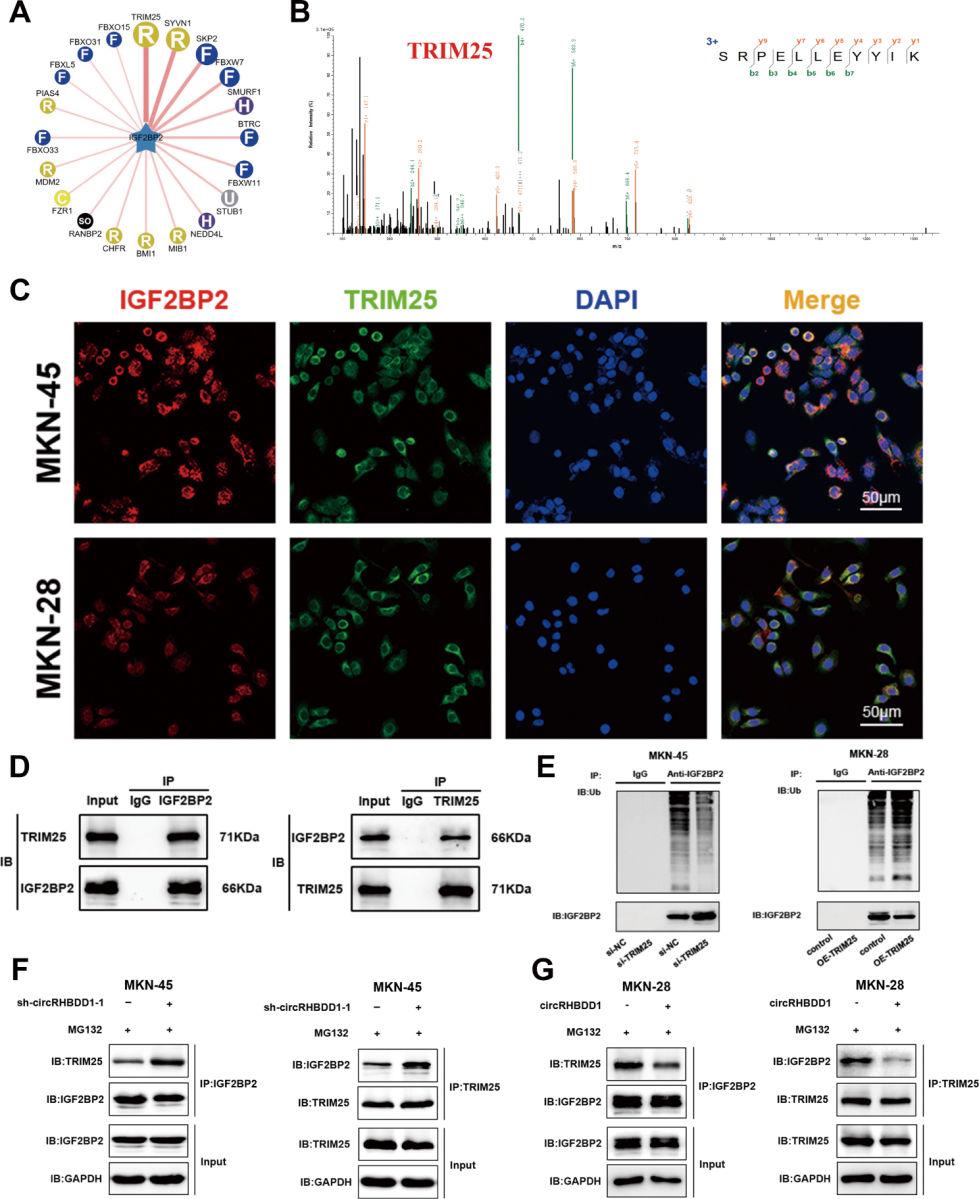
CircRHBDD1 inhibited the ubiquitination of IGF2BP2 by impeding the interaction of E3 ligase TRIM25 and IGF2BP2. **A** Prediction of the E3 ligases of IGF2BP2. **B** E3 ligase TRIM25 affecting the ubiquitination of IGF2BP2 was determined by mass spectrometry. **C** Immunofluorescence indicated that IGF2BP2 and TRIM25 were co-located in MKN-45 and MKN-28 cells. Scale bar = 50 μm. **D** Co-IP experiment revealed that IGF2BP2 interacted with TRIM25. **E** After MG-132 treatment, western blotting was used to detect the impact of TRIM25 on the ubiquitination level of IGF2BP2 in MKN-45 and MKN-28 cells. **F** Co-IP experiment showed the combination between IGF2BP2 and TRIM25 in circRHBDD1-silencing MKN-45 cells. **G** The interaction between IGF2BP2 and TRIM25 in circRHBDD1-overexpressing MKN-28 cells was detected by Co-IP experiment

Using the ubibrowser database (http://ubibrowser.ncpsb.org.cn) [33], TRIM25 was identified as a potential E3 ligase for IGF2BP2 (Fig. 5A). TRIM25, an RNA-binding protein, belongs to the E3 ubiquitin ligases of the TRIM family, which can catalyze the addition of ubiquitin chains to its substrate for degradation [34]. Mass spectrometry and immunofluorescence confirmed the interaction between TRIM25 and IGF2BP2 (Fig. 5B, C). Co-IP assays validated this interaction (Fig. 5D). TRIM25 knockdown decreased IGF2BP2 ubiquitination, while overexpression increased it (Fig. 5E). Proteasome inhibition assays showed that circRHBDD1 knockdown enhanced, while overexpression reduced, the interaction between TRIM25 and IGF2BP2 (Fig. 5F, G). These results suggest that circRHBDD1 inhibits IGF2BP2 ubiquitination by disrupting the interaction with TRIM25.

### IGF2BP2 enhanced the stability of PD-L1 mRNA via m^6^A modification in GC

**Fig. 6:**
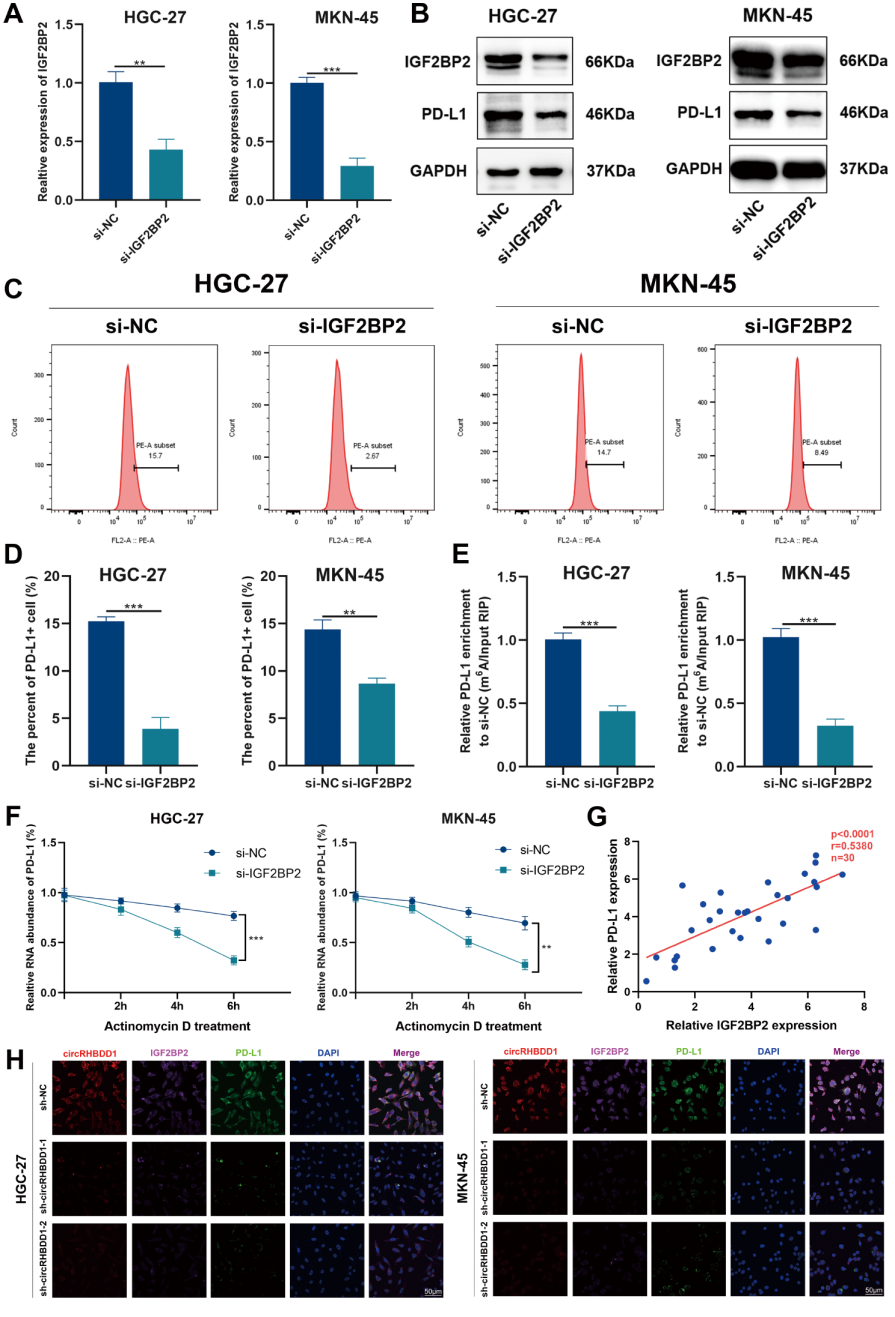
IGF2BP2 enhanced the stability of PD-L1 mRNA via m^6^A modification in GC. **A** qRT-PCR was utilized to test the knockdown level of IGF2BP2 in HGC-27 and MKN-45 cells. **B** Western blotting was utilized to test the knockdown level of IGF2BP2 in HGC-27 and MKN-45 cells and the effects of IGF2BP2 knockdown on the protein expression level of PD-L1. **C**, **D** The expression level of PD-L1 was tested by flow cytometry. **E** qRT-PCR was utilized to determine the effects of knocking down IGF2BP2 on the expression of PD-L1 m^6^A in HGC-27 and MKN-45 cells. **F** In HGC-27 and MKN-45 cells, IGF2BP2 was knocked down at indicated time points following treatment with actinomycin D. qRT-PCR was utilized to test the expression of PD-L1. **G** Pertinence assessment between IGF2BP2 and PD-L1 expression in GC tissues (*n* = 30). **H** FISH and immunofluorescence images revealing the expression levels of circRHBDD1, IGF2BP2 and PD-L1 in circRHBDD1-sliencing GC cells. Scale bar = 50 μm. Data are presented with the means ± SD of three independent experiments. **p < 0.01; ***p < 0.001

IGF2BP2, a recognized m^6^A reader, stabilizes PD-L1 mRNA [35]. Western blotting and flow cytometry showed that IGF2BP2 knockdown reduced PD-L1 protein levels (Fig. 6A-D). m^6^A quantification indicated decreased PD-L1 m^6^A levels upon IGF2BP2 knockdown (Fig. 6E). Actinomycin D assays confirmed reduced PD-L1 mRNA stability in IGF2BP2-silenced cells (Fig. 6F). Correlation analysis showed a direct association between IGF2BP2 and PD-L1 expression levels (Fig. 6G). FISH analysis demonstrated that circRHBDD1 silencing reduced IGF2BP2 and PD-L1 levels in GC cells (Fig. 6H). Rescue experiments in MKN-28 cells overexpressing circRHBDD1 showed that IGF2BP2 knockdown restored PD-L1 expression levels and T cell-mediated killing (Fig. S6A-F). These findings indicate that circRHBDD1-mediated immune escape operates through the IGF2BP2/PD-L1 axis.

### CircRHBDD1 promoted tumor growth in the models of C57BL/6 mouse

**Fig. 7:**
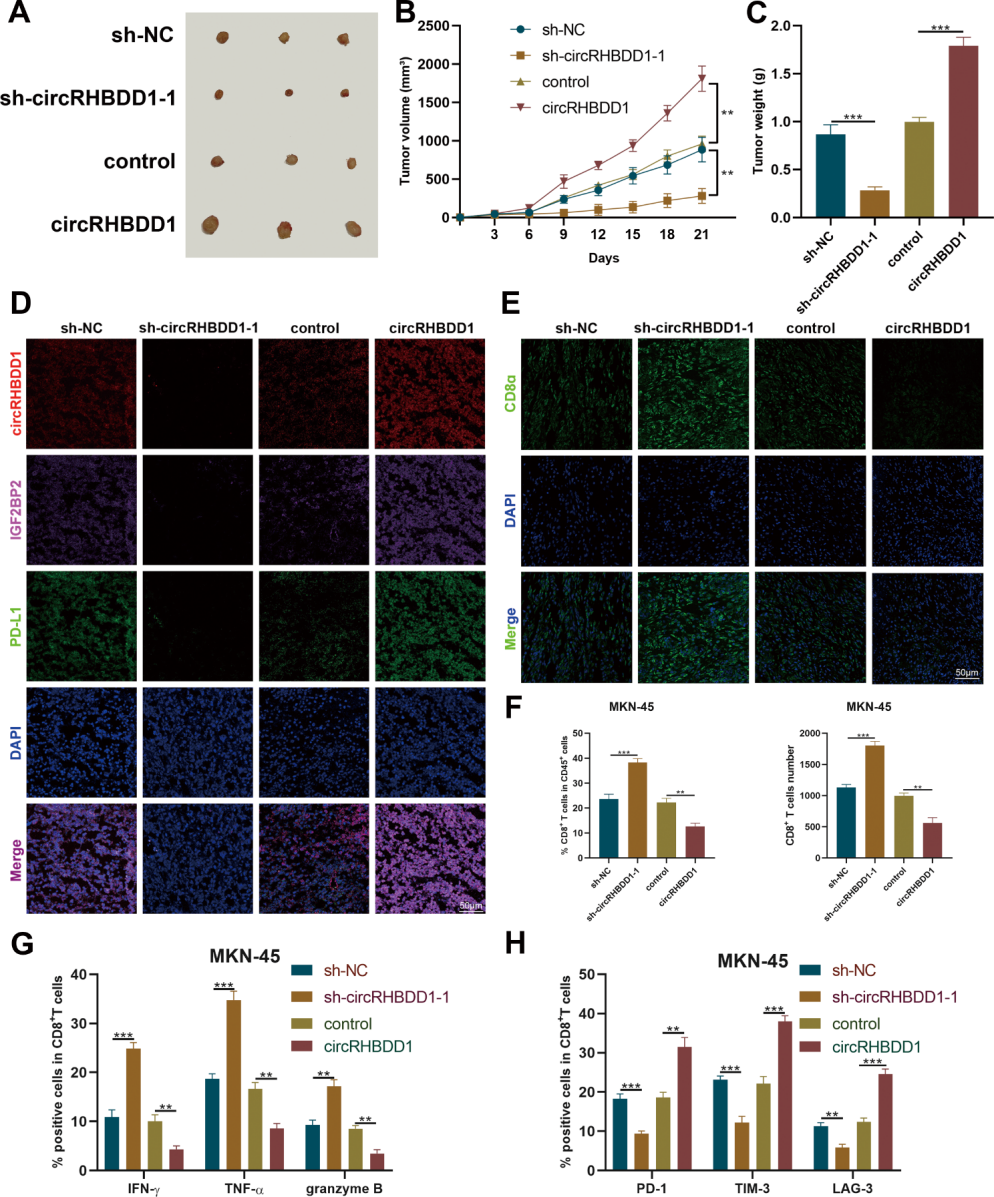
CircRHBDD1 promoted tumor growth in C57BL/6 mouse models. **A** Injecting gastric cancer cells with knocked down or overexpressed circRHBDD1 into C57BL/6 mice, and the transplanted tumor was photographed and recorded 21 days later. **B** The volume of the transplanted tumors. **C** The weight of the transplanted tumors. **D** FISH and immunofluorescence assays of circRHBDD1, IGF2BP2 and PD-L1. Scale bar = 50 μm. **E** Immunofluorescence analysis of CD8^+^ T cells. Scale bar = 50 μm. **F** Flow cytometry assays of the percentage and number of CD8^+^ T cells. **G** Presence of IFN-γ, TNF-α and granzyme B. **H** Presence of PD-1, TIM-3 and LAG-3 on the cover of CD8^+^ T cells. Data are presented with the means ± SD of three independent experiments. **p < 0.01; ***p < 0.001

To assess the role of circRHBDD1 in tumor growth in immunocompetent mice, we injected circRHBDD1-silenced or overexpressing GC cells into C57BL/6 mice. Tumor volume and weight were significantly reduced in the knockdown group and increased in the overexpression group (Fig. 7A-C). FISH and immunofluorescence confirmed that circRHBDD1 knockdown decreased, while overexpression increased, the expression of circRHBDD1, IGF2BP2, and PD-L1 (Fig. 7D). Immunofluorescence and flow cytometry indicated that circRHBDD1 knockdown promoted CD8^+^ T cell infiltration, whereas overexpression inhibited it (Fig. 7E-H). These results suggest that circRHBDD1 promotes tumor growth by modulating immune cell infiltration and activity.

### Characterization and treatment effects of PLGA-PEG(si-circRHBDD1) NPs in C57BL/6 GC models

**Fig. 8:**
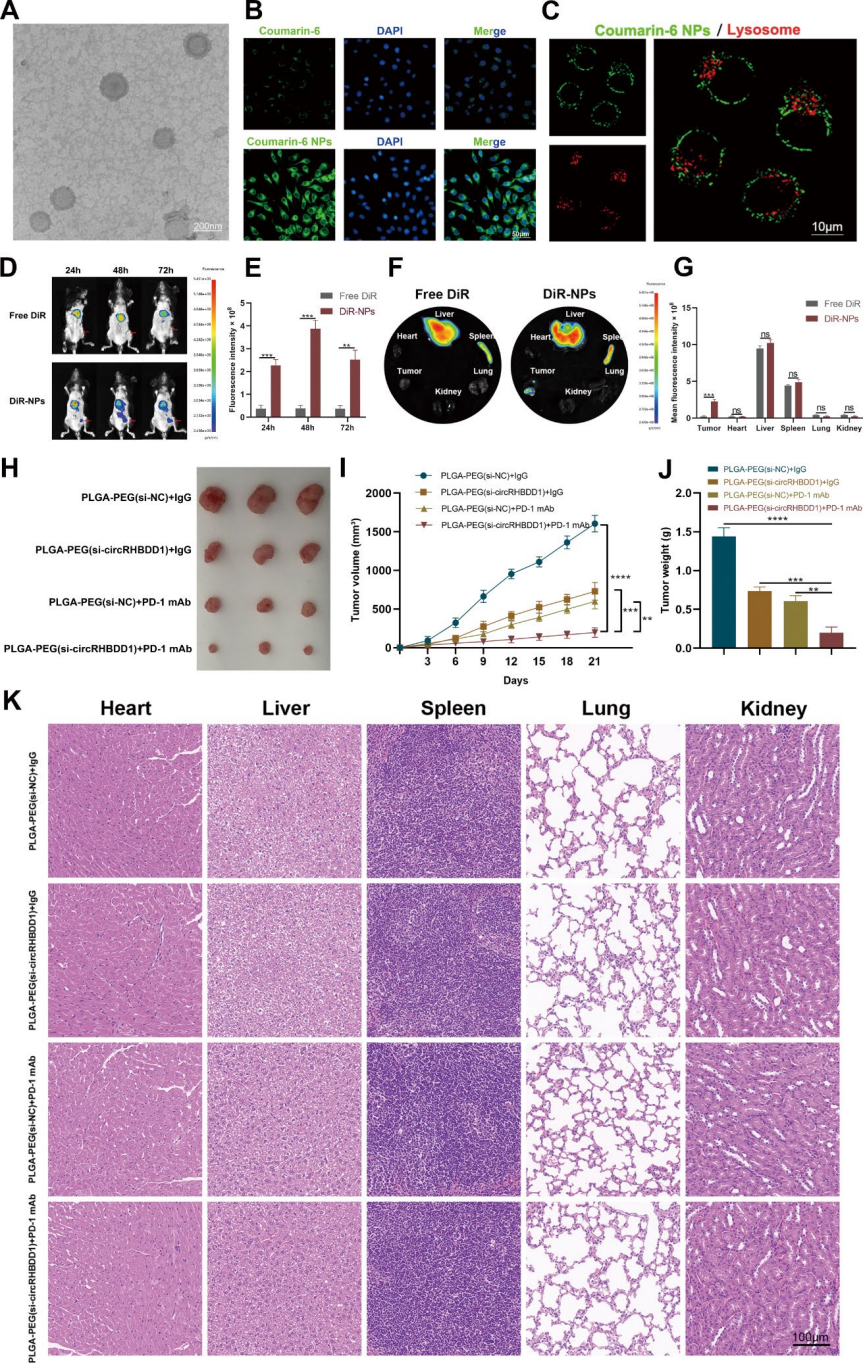
Characterization and treatment effect of PLGA-PEG(si-circRHBDD1) NPs in C57BL/6 GC models. **A** Typical TEM picture of PLGA-PEG(si-circRHBDD1) NPs. Scale bar = 200 nm. **B** Incubate MKN-28 cells with Coumarin-6 labeled NPs to evaluate the cell uptake of MKN-28 cells by NPs. Scale bar = 50 μm. **C** Cellular internalization and lysosomal escape of Coumarin-6 NPs observed by CLSM. Scale bar = 10 nm. **D**, **E** Dynamic fluorescence imaging in vivo following intravenous administration of dissociative DiR or DiR-NPs. Red arrow indicates the location of heteroplastic tumor. **F**, **G** The fluorescence images of tumor and isolated organs of dissociative DiR or DiR-NPs. **H** Validating the therapeutic effects of PLGA-PEG using a C57BL/6 mouse model. (si-circRHBDD1) combined with anti-PD-1. **I** Statistical analysis of tumor volume. **J** Analysis of tumor weight statistics. **K** HE staining of representative sections from the heart, liver, spleen, lungs, and kidneys. Scale bar = 100 μm. Data are presented with the means ± SD of three independent experiments. **p < 0.01; ***p < 0.001; ****p < 0.0001; ns not significant

The systemic delivery leads to the defect of rapid drug degradation, poor bioavailability and limited tumor site enrichment [36]. In this research, we utilized the dominance of a nano-delivery system to accurately deliver drugs to the site of tumor. PLGA-PEG(si-circRHBDD1) nanoparticles were prepared using a w/o/w emulsion method (Fig. S7A). TEM images showed spherical, uniformly dispersed NPs (Fig. 8A). DLS analysis indicated a mean diameter of 145.5 ± 2.8 nm, a zeta potential of -10.4 ± 7.62 mV, and stability in various solutions (Fig. S7B-F). In vitro release studies demonstrated controlled release of si-circRHBDD1 from PLGA-PEG NPs (Fig. S7G). Coumarin-6 NPs confirmed efficient cellular uptake and lysosomal escape (Fig. 8B, C). DiR-NPs showed enhanced tumor targeting in vivo (Fig. 8D-G). In C57BL/6 GC models, PLGA-PEG(si-circRHBDD1) combined with anti-PD-1 exhibited significant tumor growth inhibition (Fig. 8H-J) without systemic toxicity (Fig. 8K; Fig. S7H-K). These results indicate that PLGA-PEG(si-circRHBDD1) NPs combined with anti-PD-1 significantly inhibit tumor growth and are safe in vivo.

**Fig. 9:**
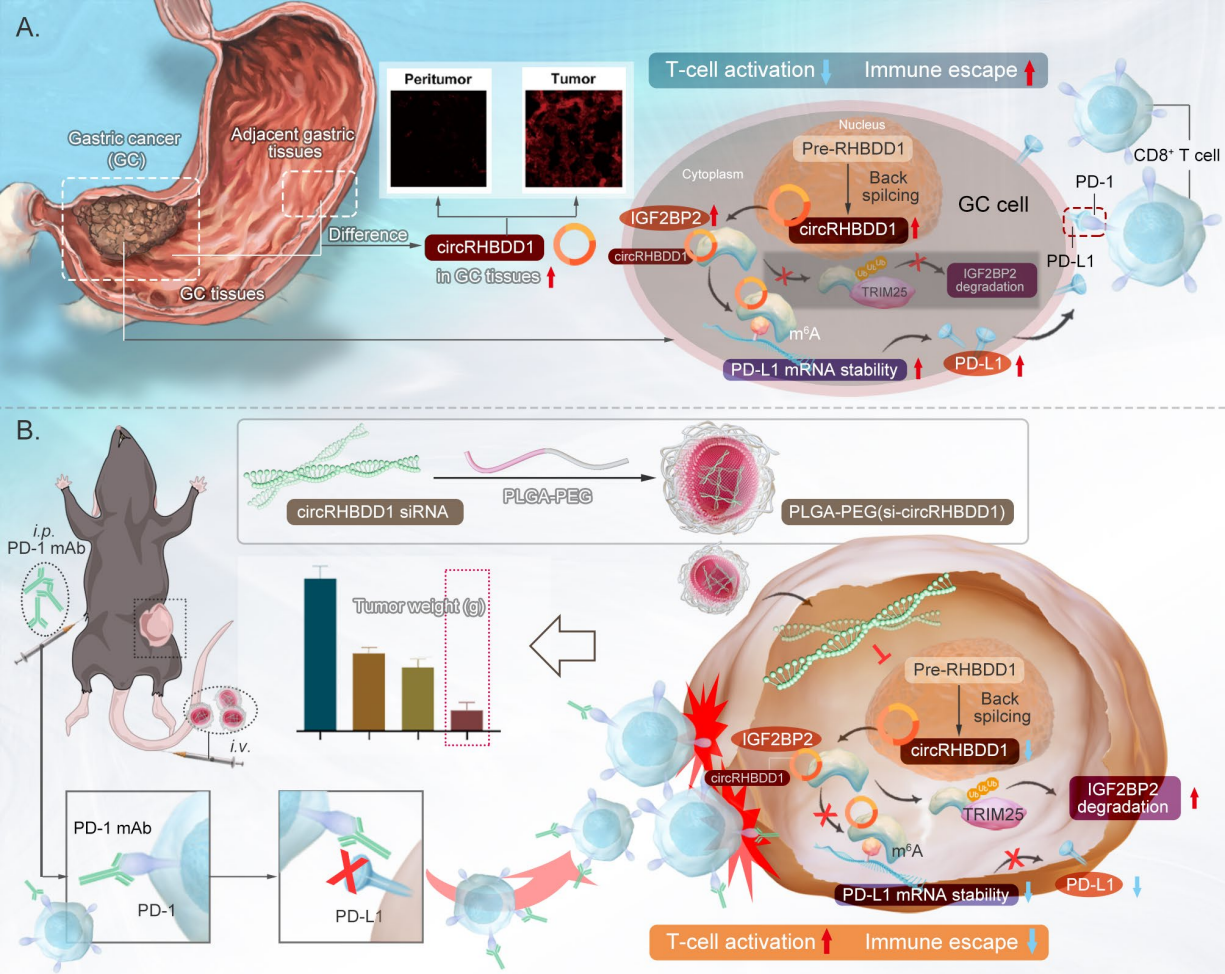
**A**, **B** Schematic diagram of circRHBDD1 in promoting immune escape through the IGF2BP2/PD-L1 axis and serving as a nanotherapeutic target in GC

Together, our study suggested that circRHBDD1 inhibited IGF2BP2 ubiquitination by competing with TRIM25, and enhanced PD-L1 mRNA stability, thereby facilitated immune evasion in GC (Fig. 9A). The combintion of PLGA-PEG(si-circRHBDD1) and anti-PD-1 could be as a new strategy for GC immunotherapy (Fig. 9B).

## Discussion

CircRNAs, a type of non-protein coding RNA formed by reverse splicing, are highly stable and widely exist in eukaryotic cytoplasm [37]. Their differential expression patterns between tumor and adjacent normal tissues suggest their potential as tumor biomarkers and drug targets [13]. With advances in high-throughput sequencing technology, many novel circRNAs have been identified in various cancers [38]. In this study, we identified circRHBDD1, formed by reverse splicing between exons 6 and 8 of RHBDD1, as highly expressed in GC tissues. High circRHBDD1 expression was associated with malignant clinicopathological features and poor prognosis. We uncovered that circRHBDD1 did not influence the proliferation capacity of GC cells in immune-deficient conditions. Our RNA sequencing analysis revealed that knockdown of circRHBDD1 led to enrichment in the PD-1/PD-L1 and immune response pathways. The interaction between PD-L1 and PD-1 induces T cell exhaustion and promotes immune escape in cancer [39]. High PD-L1 expression in tumor cells is often correlated with the clinical response to anti-PD-1/PD-L1 therapy [40]. Our findings demonstrated that circRHBDD1 upregulates PD-L1 expression, thereby enhancing immune evasion and progression of GC.

CD8^+^ T cells play a crucial role in tumor immune evasion, and their exhaustion is a major mechanism of this process [41]. Our experiments showed that knockdown of circRHBDD1 increased the percentage and number of CD8^+^ T cells in tumor tissues, along with elevated expressions of cytokines IFN-γ, TNF-α, and granzyme B, and reduced levels of coinhibitory molecules PD-1, TIM-3, and LAG-3. In vivo experiments further confirmed that circRHBDD1 knockdown inhibited tumor growth in immunocompetent C57BL/6 mice, but not in immune-deficient mice. These results suggest that circRHBDD1 knockdown exerts an anti-tumor effect by enhancing CD8^+^ T cell infiltration and activity.

CircRNAs often function as competing endogenous RNAs (ceRNAs), affecting the expression of downstream target genes [42]. They also interact with proteins, influencing their biogenesis, localization and degradation [43]. Our study found that circRHBDD1 competitively binds IGF2BP2, disrupting the interaction between TRIM25 and IGF2BP2, thereby inhibiting IGF2BP2 ubiquitination and degradation. IGF2BP2, an m^6^A ‘reader’ protein, plays a critical role in mRNA stability and tumor progression [44]. We demonstrated that IGF2BP2 enhances PD-L1 mRNA stability via m^6^A modification, contributing to immune escape in GC. In NSCLC, circIGF2BP3 has been found to facilitate tumor immune evasion by promoting the deubiquitination of PD-L1 [45]. circ-0000512 inhibited PD-L1 ubiquitination through regulating the miR-622/CMTM6 axis, thus aggravating TNBC progression and immune escape [46]. However, there is limited understanding regarding the involvement of circRNA in tumor immunity of GC. Our findings indicated that circRHBDD1 facilitated GC immune evasion by upregulating PD-L1 expression and suppressing CD8^+^ T cell infiltration. Therefore, our study revealed a novel molecular mechanism of circRHBDD1 in promoting GC immune escape via the IGF2BP2/PD-L1 axis.

Targeted siRNA delivery using nanoparticles is a promising approach in cancer therapy [47]. Traditional delivery methods such as liposomes and viral vectors have limitations, including toxicity and poor sustained release [48]. PLGA-PEG nanoparticles, approved by the FDA, offer advantages such as non-toxicity, biodegradability, prolonged circulation time, and controlled release [49]. Our study developed PLGA-PEG nanoparticles for delivering si-circRHBDD1. The nanoparticles exhibited controlled release, enhanced cellular uptake, and strong tumor-targeting ability in vivo. Importantly, PLGA-PEG(si-circRHBDD1) combined with anti-PD-1 significantly inhibited tumor growth in C57BL/6 models without obvious toxic side effects, suggesting its feasibility and safety as a therapeutic strategy.

In this study, our study identified circRHBDD1 as a key regulator of immune escape in GC by upregulating PD-L1 expression and interacting with IGF2BP2 to stabilize PD-L1 mRNA. Knockdown of circRHBDD1 enhanced CD8^+^ T cell infiltration and activity, exerting an anti-tumor effect. Moreover, our data demonstrated that the combination of PLGA-PEG(si-circRHBDD1) and anti-PD-1 significantly impeded tumor growth in C57BL/6 mouse models. This robust evidence validates that nanoparticle-mediated delivery of si-circRHBDD1 sensitizes GC cells to anti-PD-1 immunotherapy, suggesting that the nanodrug has a great potential for the clinical treatment of GC patients.

Importantly, PLGA-PEG NPs used in this study are FDA-approved, safe and reliable in clinic. However, they may have several limitations. One potential drawback is the reliance on animal models, which may not fully replicate human cancer biology. This issue could be addressed through increasing emphasis on clinical trials in the future. Additionally, the long-term effects and potential off-target effects of the nanoparticle delivery system were not extensively covered, so it also needs to improved nanoparticle formulations. Furthermore, it is essential to investigate the control of drug release rate within the body and the biodegradation mechanism. Addressing the prolonged duration required for clinical trials remains a critical challenge. Overcoming these challenges will help to bring new therapeutic options to patients. This work offered valuable clues for combining PLGA-PEG(si-circRHBDD1) and anti-PD-1 for the potential clinical therapy of GC. Moreover, this combined immunotherapy has the potential of wider application in other cancer types.

## Conclusions

In summary, our study identifies circRHBDD1 as a critical regulator of immune escape in gastric cancer. The upregulation of circRHBDD1 correlates with poor patient prognosis and promotes tumor immune evasion by enhancing PD-L1 expression and reducing CD8^+^ T cell infiltration. Mechanistically, circRHBDD1 prevents the degradation of IGF2BP2, which stabilizes PD-L1 mRNA through m^6^A modification, thereby facilitating immune escape. These findings highlight circRHBDD1 as a promising therapeutic target. The PLGA-PEG nanoparticle-based delivery system of si-circRHBDD1 can be used as a promising treatment to enhance the efficacy of anti-PD-1 immunotherapy without prominent systemic side effects, which needs to be further investigated in future clinical applications.

### Electronic supplementary material

Below is the link to the electronic supplementary material.

Supplementary Material 1

Supplementary Material 2

Supplementary Material 3

Supplementary Material 4

Supplementary Material 5

Supplementary Material 6

Supplementary Material 7

Supplementary Material 8

Supplementary Material 9

Supplementary Material 10

Supplementary Material 11

## Data Availability

The data sets used to support the findings of this study are available from the corresponding author on reasonable request.

## References

[1] Sung H, Ferlay J, Siegel RL, Laversanne M, Soerjomataram I, Jemal A, et al. Global Cancer statistics 2020: GLOBOCAN estimates of incidence and Mortality Worldwide for 36 cancers in 185 countries. CA Cancer J Clin. 2021;713:209–49. 10.3322/caac.21660 33538338

[2] Ajani JA, D’Amico TA, Bentrem DJ, Chao J, Cooke D, Corvera C, et al. Gastric Cancer, Version 2.2022, NCCN Clinical Practice guidelines in Oncology. J Natl Compr Canc Netw. 2022;202:167–92. 10.6004/jnccn.2022.0008 35130500

[3] Ferlay J, Colombet M, Soerjomataram I, Parkin DM, Piñeros M, Znaor A, et al. Cancer statistics for the year 2020: an overview. Int J Cancer. 2021;149:778–89. 10.1002/ijc.33588 33818764

[4] Ilic M, Ilic I. Epidemiology of stomach cancer. World J Gastroenterol. 2022;2812:1187–203. 10.3748/wjg.v28.i12.1187 PMC8968487 35431510

[5] Wang S, Zheng R, Li J, Zeng H, Li L, Chen R, et al. Global, regional, and national lifetime risks of developing and dying from gastrointestinal cancers in 185 countries: a population-based systematic analysis of GLOBOCAN. Lancet Gastroenterol Hepatol. 2024;93:229–37. 10.1016/S2468-1253(23)00366-7 PMC10849975 38185129

[6] Jiang Y, Zhang Q, Hu Y, Li T, Yu J, Zhao L, et al. ImmunoScore signature: a Prognostic and Predictive Tool in Gastric Cancer. Ann Surg. 2018;2673:504–13. 10.1097/SLA.0000000000002116 28002059

[7] Sitarz R, Skierucha M, Mielko J, Offerhaus GJA, Maciejewski R, Polkowski WP. Gastric cancer: epidemiology, prevention, classification, and treatment. Cancer Manag Res. 2018;10:239–48. 29445300 10.2147/CMAR.S149619 PMC5808709

[8] Guan WL, He Y, Xu RH. Gastric cancer treatment: recent progress and future perspectives. J Hematol Oncol. 2023;161:57. 10.1186/s13045-023-01451-3 PMC10225110 37245017

[9] Liu CX, Chen LL. Circular RNAs: characterization, cellular roles, and applications. Cell. 2022;18512:2016–34. 10.1016/j.cell.2022.04.021 35584701

[10] Salami R, Salami M, Mafi A, Vakili O, Asemi Z. Circular RNAs and glioblastoma multiforme: focus on molecular mechanisms. Cell Commun Signal. 2022;201:13. 10.1186/s12964-021-00809-9 PMC8796413 35090496

[11] Xue C, Li G, Lu J, Li L. Crosstalk between circRNAs and the PI3K/AKT signaling pathway in cancer progression. Signal Transduct Target Ther. 2021;61:400. 10.1038/s41392-021-00788-w PMC8611092 34815385

[12] Lei M, Zheng G, Ning Q, Zheng J, Dong D. Translation and functional roles of circular RNAs in human cancer. Mol Cancer. 2020;191:30. 10.1186/s12943-020-1135-7 PMC7023758 32059672

[13] Li Z, Cheng Y, Fu K, Lin Q, Zhao T, Tang W, et al. Circ-PTPDC1 promotes the progression of gastric Cancer through sponging Mir-139-3p by regulating ELK1 and functions as a prognostic biomarker. Int J Biol Sci. 2021;1715:4285–304. 10.7150/ijbs.62732 PMC8579456 34803498

[14] Kristensen LS, Jakobsen T, Hager H, Kjems J. The emerging roles of circRNAs in cancer and oncology. Nat Rev Clin Oncol. 2022;193:188–206. 10.1038/s41571-021-00585-y 34912049

[15] Zhao R, Ni J, Lu S, Jiang S, You L, Liu H, et al. CircUBAP2-mediated competing endogenous RNA network modulates tumorigenesis in pancreatic adenocarcinoma. Aging. 2019;1119:8484–501. 10.18632/aging.102334 PMC6814619 31584877

[16] Pei X, Chen SW, Long X, Zhu SQ, Qiu BQ, Lin K, et al. circMET promotes NSCLC cell proliferation, metastasis, and immune evasion by regulating the miR-145-5p/CXCL3 axis. Aging. 2020;1213:13038–58. 10.18632/aging.103392 PMC7377868 32614785

[17] Li K, Zhang A, Li X, Zhang H, Zhao L. Advances in clinical immunotherapy for gastric cancer. Biochim Biophys Acta Rev Cancer. 2021;18762:188615. 10.1016/j.bbcan.2021.188615 34403771

[18] Bashash D, Zandi Z, Kashani B, Pourbagheri-Sigaroodi A, Salari S, Ghaffari SH. Resistance to immunotherapy in human malignancies: mechanisms, research progresses, challenges, and opportunities. J Cell Physiol. 2022;2371:346–72. 10.1002/jcp.30575 34498289

[19] Majumder J, Taratula O, Minko T. Nanocarrier-based systems for targeted and site specific therapeutic delivery. Adv Drug Deliv Rev. 2019;144:57–77. 31400350 10.1016/j.addr.2019.07.010 PMC6748653

[20] Rosic G, Selakovic D, Omarova S, CANCER SIGNALING, CELL/GENE THERAPY, DIAGNOSIS AND ROLE OF NANOBIOMATERIALS. Adv Biology Earth Sci. 2024;9:11–34. 10.62476/abes9s11

[21] Huseynov E, Khalilov R, Mohamed AJ, NOVEL NANOMATERIALS FOR HEPATOBILIARY, DISEASES TREATMENT AND FUTURE PERSPECTIVES. Adv Biology Earth Sci. 2024;9:81–91. 10.62476/abes9s81

[22] Gu P, Wusiman A, Wang S, Zhang Y, Liu Z, Hu Y, et al. Polyethylenimine-coated PLGA nanoparticles-encapsulated Angelica Sinensis polysaccharide as an adjuvant to enhance immune responses. Carbohydr Polym. 2019;223:115128. 31427012 10.1016/j.carbpol.2019.115128

[23] Yin T, Fan Q, Hu F, Ma X, Yin Y, Wang B, et al. Engineered macrophage-membrane-coated nanoparticles with enhanced PD-1 expression induce Immunomodulation for a synergistic and targeted Antiglioblastoma Activity. Nano Lett. 2022;2216:6606–14. 10.1021/acs.nanolett.2c01863 35948420

[24] Bai X, Zhao G, Chen Q, Li Z, Gao M, Ho W, et al. Inhaled siRNA nanoparticles targeting IL11 inhibit lung fibrosis and improve pulmonary function post-bleomycin challenge. Sci Adv. 2022;825:eabn7162. 10.1126/sciadv.abn7162 PMC9216512 35731866

[25] Nagaraju GP, Srivani G, Dariya B, Chalikonda G, Farran B, Behera SK, et al. Nanoparticles guided drug delivery and imaging in gastric cancer. Semin Cancer Biol. 2021;69:69–76. 31954835 10.1016/j.semcancer.2020.01.006

[26] Wei PS, Chen YJ, Lin SY, Chuang KH, Sheu MT, Ho HO. In situ subcutaneously injectable thermosensitive PEG-PLGA diblock and PLGA-PEG-PLGA triblock copolymer composite as sustained delivery of bispecific anti-CD3 scFv T-cell/anti-EGFR Fab Engager (BiTEE). Biomaterials. 2021;278:121166. 34634663 10.1016/j.biomaterials.2021.121166

[27] Li CW, Lim SO, Xia W, Lee HH, Chan LC, Kuo CW, et al. Glycosylation and stabilization of programmed death ligand-1 suppresses T-cell activity. Nat Commun. 2016;7:12632. 27572267 10.1038/ncomms12632 PMC5013604

[28] Wang L, Griffel B, Xu X. Synthesis of PLGA-Lipid hybrid nanoparticles for siRNA delivery using the Emulsion Method PLGA-PEG-Lipid nanoparticles for siRNA delivery. Methods Mol Biol. 2017;1632:231–40. 28730443 10.1007/978-1-4939-7138-1_15

[29] Yi M, Niu M, Xu L, Luo S, Wu K. Regulation of PD-L1 expression in the tumor microenvironment. J Hematol Oncol. 2021;141:10. 10.1186/s13045-020-01027-5 PMC7792099 33413496

[30] Chow A, Perica K, Klebanoff CA, Wolchok JD. Clinical implications of T cell exhaustion for cancer immunotherapy. Nat Rev Clin Oncol. 2022;1912:775–90. 10.1038/s41571-022-00689-z PMC10984554 36216928

[31] Li JH, Liu S, Zhou H, Qu LH, Yang JH. starBase v2.0: decoding miRNA-ceRNA, miRNA-ncRNA and protein-RNA interaction networks from large-scale CLIP-Seq data. Nucleic Acids Res. 2014;42Database issue:D92–7. 10.1093/nar/gkt1248 PMC3964941 24297251

[32] Armaos A, Colantoni A, Proietti G, Rupert J, Tartaglia GG. catRAPID omics v2.0: going deeper and wider in the prediction of protein-RNA interactions. Nucleic Acids Res. 2021;49W1:W72–9. 10.1093/nar/gkab393 PMC8262727 34086933

[33] Wang X, Li Y, He M, Kong X, Jiang P, Liu X, et al. UbiBrowser 2.0: a comprehensive resource for proteome-wide known and predicted ubiquitin ligase/deubiquitinase-substrate interactions in eukaryotic species. Nucleic Acids Res. 2022;50D1:D719–28. 10.1093/nar/gkab962 PMC8728189 34669962

[34] Mei P, Xie F, Pan J, Wang S, Gao W, Ge R, et al. E3 ligase TRIM25 ubiquitinates RIP3 to inhibit TNF induced cell necrosis. Cell Death Differ. 2021;2810:2888–99. 10.1038/s41418-021-00790-3 PMC8481267 33953350

[35] Zhang Z, Xing Y, Gao W, Yang L, Shi J, Song W, et al. N(6)-methyladenosine (m(6)A) reader IGF2BP2 promotes gastric cancer progression via targeting SIRT1. Bioengineered. 2022;135:11541–50. 10.1080/21655979.2022.2068920 PMC9275927 35502827

[36] Jiang H, Wang Q, Li L, Zeng Q, Li H, Gong T, et al. Turning the Old Adjuvant from Gel to nanoparticles to amplify CD8(+) T cell responses. Adv Sci (Weinh). 2018;51:1700426. 10.1002/advs.201700426 PMC5770685 29375970

[37] Goodall GJ, Wickramasinghe VO. RNA in cancer. Nat Rev Cancer. 2021;211:22–36. 10.1038/s41568-020-00306-0 33082563

[38] Chen S, Huang V, Xu X, Livingstone J, Soares F, Jeon J, et al. Widespread and functional RNA circularization in localized prostate Cancer. Cell. 2019;1764:831–e843822. 10.1016/j.cell.2019.01.025 30735634

[39] Filippone A, Lanza M, Mannino D, Raciti G, Colarossi C, Sciacca D, et al. PD1/PD-L1 immune checkpoint as a potential target for preventing brain tumor progression. Cancer Immunol Immunother. 2022;719:2067–75. 10.1007/s00262-021-03130-z PMC9374620 35092481

[40] Dai X, Liu J, Wei W. Mitochondrial PD-L1 modulates cancer immunotherapy. Cell Res. 2023;335:335–6. 10.1038/s41422-023-00777-4 PMC10156688 36693901

[41] Dolina JS, Van Braeckel-Budimir N, Thomas GD, Salek-Ardakani S. CD8(+) T cell exhaustion in Cancer. Front Immunol. 2021;12:715234. 34354714 10.3389/fimmu.2021.715234 PMC8330547

[42] Liang M, Yao W, Shi B, Zhu X, Cai R, Yu Z, et al. Circular RNA hsa_circ_0110389 promotes gastric cancer progression through upregulating SORT1 via sponging mir-127-5p and miR-136-5p. Cell Death Dis. 2021;127:639. 10.1038/s41419-021-03903-5 PMC8222372 34162830

[43] Okholm TLH, Sathe S, Park SS, Kamstrup AB, Rasmussen AM, Shankar A, et al. Transcriptome-wide profiles of circular RNA and RNA-binding protein interactions reveal effects on circular RNA biogenesis and cancer pathway expression. Genome Med. 2020;121:112. 10.1186/s13073-020-00812-8 PMC7722315 33287884

[44] Liu Y, Shi M, He X, Cao Y, Liu P, Li F, et al. LncRNA-PACERR induces pro-tumour macrophages via interacting with mir-671-3p and m6A-reader IGF2BP2 in pancreatic ductal adenocarcinoma. J Hematol Oncol. 2022;151:52. 10.1186/s13045-022-01272-w PMC9077921 35526050

[45] Liu Z, Wang T, She Y, Wu K, Gu S, Li L, et al. N(6)-methyladenosine-modified circIGF2BP3 inhibits CD8(+) T-cell responses to facilitate tumor immune evasion by promoting the deubiquitination of PD-L1 in non-small cell lung cancer. Mol Cancer. 2021;201:105. 10.1186/s12943-021-01398-4 PMC8377850 34416901

[46] Dong LF, Chen FF, Fan YF, Zhang K, Chen HH. circ-0000512 inhibits PD-L1 ubiquitination through sponging miR-622/CMTM6 axis to promote triple-negative breast cancer and immune escape. J Immunother Cancer. 2023;116. 10.1136/jitc-2022-005461 PMC10314703 37349124

[47] El-Say KM, El-Sawy HS. Polymeric nanoparticles: promising platform for drug delivery. Int J Pharm. 2017;5281–2:675–91. 10.1016/j.ijpharm.2017.06.052 28629982

[48] Sousa AR, Oliveira AV, Oliveira MJ, Sarmento B. Nanotechnology-based siRNA delivery strategies for metastatic colorectal cancer therapy. Int J Pharm. 2019;568:118530. 31323369 10.1016/j.ijpharm.2019.118530

[49] Dawidczyk CM, Kim C, Park JH, Russell LM, Lee KH, Pomper MG, et al. State-of-the-art in design rules for drug delivery platforms: lessons learned from FDA-approved nanomedicines. J Control Release. 2014;187:133–44. 24874289 10.1016/j.jconrel.2014.05.036 PMC4132889

