# Supplementary material for: CircRHBDD1 promotes immune escape via IGF2BP2/PD-L1 signaling and acts as a nanotherapeutic target in gastric cancer
Source: J Transl Med. 2024 Jul 30;22:704. doi: 10.1186/s12967-024-05498-9 (PMC11289934; doi:10.1186/s12967-024-05498-9)
Supplement: Supplementary file 1 — Supplementary Material 1 [file 12967_2024_5498_MOESM1_ESM.docx]

**Table S1.** The correlation between clinicopathological characteristics and circRHBDD1 expression level in 45 gastric cancer patients.

| **Characteristics** |  | **circRHBDD1 expression** | | ***P*** |
| --- | --- | --- | --- | --- |
|  |  | **Low** | **High** |  |
| Gender | Female | 5 | 5 | 0.968 |
|  | Male | 20 | 15 |  |
| Age | <60 years | 4 | 7 | 0.261 |
|  | ≥60 years | 21 | 13 |  |
| Differentiation | Well | 14 | 20 | **0.002** |
|  | Moderate/Poor | 11 | 0 |  |
| Neural invasion | No | 11 | 4 | 0.168 |
|  | Yes | 14 | 16 |  |
| Vascular invasion | No | 15 | 8 | 0.182 |
|  | Yes | 10 | 12 |  |
| Lymph invasion | No | 13 | 8 | 0.423 |
|  | Yes | 12 | 12 |  |
| Tumor location | Middle/down | 20 | 15 | 0.968 |
|  | Upper | 5 | 5 |  |
| Tumor size | < 5 cm | 21 | 9 | **0.015** |
|  | ≥ 5 cm | 4 | 11 |  |
| AJCC stage | I/II | 16 | 9 | 0.202 |
|  | III/IV | 9 | 11 |  |
