# Supplementary material for: CircRHBDD1 promotes immune escape via IGF2BP2/PD-L1 signaling and acts as a nanotherapeutic target in gastric cancer
Source: J Transl Med. 2024 Jul 30;22:704. doi: 10.1186/s12967-024-05498-9 (PMC11289934; doi:10.1186/s12967-024-05498-9)
Supplement: Supplementary file 2 — Supplementary Material 2 [file 12967_2024_5498_MOESM2_ESM.docx]

**Table S2.** Univariate and multivariable analysis of overall survival in patients with gastric cancer.

| **Variables** | **Univariate Multivariate** | | | |
| --- | --- | --- | --- | --- |
|  | **Log-rank** | ***P*** | **HR (95% CI)** | ***P*** |
| Gender (Male vs Female) | 0.474 | 0.491 |  |  |
| Age (≥ 60 years *vs* < 60 years) | 2.374 | 0.123 |  |  |
| Differentiation (Moderate/Poor *vs* Well) | 5.482 | 0.019 | Not included |  |
| Neural invasion (Yes *vs* No) | 2.774 | 0.096 |  |  |
| Vascular invasion (Yes *vs* No) | 0.371 | 0.542 |  |  |
| Lymph invasion (Yes *vs* No) | 3.189 | 0.074 |  |  |
| Tumor location (Upper *vs* middle/down) | 2.697 | 0.101 |  |  |
| Tumor size (≥ 5 cm *vs* < 5 cm) | 15.883 | < 0.001 | 4.093(1.447-11.574) | 0.008 |
| AJCC stage (III/IV *vs* I/II) | 12.082 | 0.001 | 5.523(1.547-19.713) | 0.008 |
| CircRHBDD1 expression (High *vs* Low) | 8.249 | 0.004 | 3.307(1.134-9.647) | 0.029 |
