# Supplementary material for: CircRHBDD1 promotes immune escape via IGF2BP2/PD-L1 signaling and acts as a nanotherapeutic target in gastric cancer
Source: J Transl Med. 2024 Jul 30;22:704. doi: 10.1186/s12967-024-05498-9 (PMC11289934; doi:10.1186/s12967-024-05498-9)
Supplement: Supplementary file 3 — Supplementary Material 3 [file 12967_2024_5498_MOESM3_ESM.docx]

**Table S3.** Univariate and multivariable analysis of disease-free survival in patients with gastric cancer.

| **Variables** | **Univariate Multivariate** | | | |
| --- | --- | --- | --- | --- |
|  | **Log-rank** | ***P*** | **HR (95% CI)** | ***P*** |
| Gender (Male vs Female) | 0.500 | 0.479 |  |  |
| Age (≥ 60 years *vs* < 60 years) | 1.362 | 0.243 |  |  |
| Differentiation (Moderate/Poor *vs* Well) | 4.281 | 0.039 | Not included |  |
| Neural invasion (Yes *vs* No) | 3.191 | 0.074 |  |  |
| Vascular invasion (Yes *vs* No) | 1.085 | 0.298 |  |  |
| Lymph invasion (Yes *vs* No) | 6.258 | 0.012 | Not included |  |
| Tumor location (Upper *vs* middle/down) | 2.431 | 0.119 |  |  |
| Tumor size (≥ 5 cm *vs* < 5 cm) | 13.338 | < 0.001 | 3.182(1.112-9.105) | 0.031 |
| AJCC stage (III/IV *vs* I/II) | 14.376 | < 0.001 | 9.425(2.329-38.140) | 0.002 |
| CircRHBDD1 expression (High *vs* Low) | 8.120 | 0.004 | 4.998(1.442-17.324) | 0.011 |
