# Supplementary material for: CircRHBDD1 promotes immune escape via IGF2BP2/PD-L1 signaling and acts as a nanotherapeutic target in gastric cancer
Source: J Transl Med. 2024 Jul 30;22:704. doi: 10.1186/s12967-024-05498-9 (PMC11289934; doi:10.1186/s12967-024-05498-9)
Supplement: Supplementary file 4 — Supplementary Material 4 [file 12967_2024_5498_MOESM4_ESM.docx]

**SUPPLEMENTARY METHODS**

**CircRNA sequencing**

CircRNA sequencing using three paired GC tissues and adjacent gastric tissues was performed by RiboBio (Guangzhou, China). The total RNA was extracted using TRIzol reagent (Invitrogen, Carlsbad, CA, USA). Evaluation of RNA purity was conducted by ND-1000 Nanodrop, and detection of RNA integrity was carried out by Agilent 2200 TapeStation (Agilent Technologies, Santa Clara, CA, USA). Then, RNA was puriﬁed by rRNA depletion, followed by cDNA synthesis and sequencing. CircRNAs were identified by CIRI2 and CIRCexplorer2 algorithms. Based on the software package DESeq2, the differentially expressed circRNAs were determined (*p* < 0.05 and the fold change > 2).

**Cell culture**

Human gastric mucosal epithelial cell line GES-1 and GC cell lines HGC-27, AGS were purchased from American Type Culture Collection (Manassas, VA, USA), MKN-45, MKN-28 cell lines were purchased from JCRB Cell Bank (National Institute of Hygienic Sciences, Tokyo, Japan). The mouse GC cell line MFC were purchased from Guangzhou Saiku Biotechnology (Guangzhou, China). All cell lines were cultured in RPMI-1640 medium (Gibco, CA, USA), supplemented with 10% fetal bovine serum (FBS) and 1% penicillin-streptomycin. Cells were maintained at 37°C with 5% CO_2_ in a humidified incubator. The cells were incubated with cyclohexamide (CHX) (50 µg/ml) at the specified time (0, 24, 48, 72 h), and treated with MG-132 (20 µM) or chloroquine (CQ, 20 µM) to inhibit protein synthesis or degradation.

**Lentivirus transfection and stable cell line construction**

HGC-27 and MKN-45 cells were transfected with short hairpin RNA (shRNA) against circRHBDD1 (sh-circRHBDD1-1 and sh-circRHBDD1-2) and the corresponding control (sh-NC). shRNAs and plasmids were purchased from GenePharma (Shanghai, China). The shRNA sequences were as follows: sh-circRHBDD1-1: 5’-TGGGACCGAGGACTTCCTT-3’, sh-circRHBDD1-2: 5’-GGACCGAGGACTTCCTTCG-3’.

**Plasmids and siRNAs transfection**

The plasmids overexpressing circRHBDD1 or TRIM25, and siRNAs targeting IGF2BP2 or TRIM25 were designed and synthesized by GenePharma (Shanghai, China). Cells were seeded into 6-well plates to 60%-70% confluence, and cells were transfected based on the manufacturer’s instructions of Lipofectamine 3000 reagents (Invitrogen). The siRNA sequences were as follows: si-IGF2BP2:5’-GCCGCAUGAUUCUUGAAAUTT-3’, si-TRIM25: 5’-CCUCGACAAGGAAGAUAAATT-3’.

**RNA isolation, reverse transcription, and** **quantitative real-time PCR (qRT-PCR)**

Total RNA was extracted from tissues and cells using TRIzol Universal (TIANGEN, Beijing, China). The expression levers of circRHBDD1 were analyzed by Reverse Transcription Prime and 2 × qPCR SYBR Green Master Mix kit (GENESEED, Guangzhou, China). For the detection of PD-L1 and IGF2BP2 expression, the extracted RNA was reverse transcribed into cDNA using 5 × FastKing-RT SuperMix (TIANGEN, Beijing, China). qRT-PCR was performed using the 2 × SuperReal PreMix Plus (SYBR Green; TIANGEN). The levels of circRNAs and mRNAs were normalized using GAPDH. CircRHBDD1 primers were purchased from RiboBio (Guangzhou, China). PD-L1, IGF2BP2and GAPDH were designed and synthesized by GenePharma (Shanghai, China). The primers used for qRT-PCR are shown as follows: PD-L1: forward: 5’-GGTAAGACCACCACCACCAAT-3’, reverse: 5’-TGATTCTCAGTGTGCTGGTCAC-3’; IGF2BP2: forward: 5’-GGCTTGGTTGGAAGACTGATT-3’, reverse: 5’-TGCCCTTCACAGTGATGGTT-3’; GAPDH: forward: 5’-AATCCCATCACCATCTTCC-3’, reverse: 5’-CATCACGCCACAGTTTCC-3’.

**RNA stability assay**

GC cells were incubated with actinomycin D (2 mg/ml) at 37°C with 5% CO_2_ to assess the stability of circRHBDD1 and its linear isoform mRNA stability. Total RNA was extracted from cells at indicated time points.

**RNase R treatment**

Total RNA of the indicated cells was treated with or without RNase R (5 U/μg RNA) for 37℃ at 15 min. After digestion, the RNA was subjected to subsequent experiments.

**Nuclear and cytoplasmic fractions assay**

The cytoplasmic and nuclear RNA purification kit (AmyJet scientific, Wuhan, China) was employed to extract nuclear and cytoplasmic RNA in cells according to the manufacturer’s protocol. qRT-PCR was used to detect the relative expression of circRHBDD1 in the nuclear and cytoplasmic fractions.

**Cell Counting Kit-8 assay**

GC cells were seeded in 96-well plates (2 × 10^3^ cells/well) and cultured in 100 μl of RPMI-1640 culture medium containing 10% FBS and 1% penicillin-streptomycin. After that, we added 10 μl of CCK-8 solution (Bestbio, Shanghai, China) to each well at indicated time points (0, 24, 48, 72 h) according to the manufacturer’s protocol. After 2 h incubation in the dark, the absorbance value at 450 nm was examined (BioTek).

**Colony formation assay**

GC Cells were seeded in 6-well plates at 37°C with 5% CO_2_ for 2 weeks. Then, these cells were washed three times with PBS and fixed with 4% paraformaldehyde for 25 min and then stained with crystal violet staining solution for 30 min.

**5-Ethynyl-2’-deoxyuridine (EdU) assay**

GC cells were seeded in 96-well plates (1.5 × 10^4^ cells/well) at 37°C with 5% CO_2_ for 1 d. After reaching 90% confluency, a Cell-Light EdU DNA Cell Proliferation Kit (RiboBio) were used based on the manufacturer’s instructions. After incubation with EdU for 2 h, the GC cells were fixed with 4% paraformaldehyde and stained with Apollo Dye Solution. Then the cells were stained with Hoechst 33342.

**RNA sequencing analysis**

Total RNA was isolated from sh-NC (n = 3) and sh-circRHBDD1-1 (n = 3) HGC-27 cells. RNA samples were performed with high-throughput sequencing (BGI, Shenzhen, China). Briefly, BGISEQ-500 platform was used to sequence the samples. According to the fold changes > 2.0 and *p* < 0.001, the differential mRNA abundance was analyzed by DESeq2.

**Western blotting analysis**

RIPA Lysis Buffer and PMSF were mixed in a ratio of 100:1 and used to extract total protein from cells. Then we used BCA assay to determine the protein concentrations. The protein was separated by 10% SDS-PAGE, transferred to PVDF membrane (Millipore, CA, USA) and blocked with Quickblock Blocking Buffer with TBST for 20 min. Next, the membranes were incubated with primary antibodies at 4°C overnight. Primary antibodies including anti-PD-L1 antibody (CST, USA), anti-IGF2BP2 antibody (Abcam, UK), anti-ubiquitin antibody (CST, USA), anti-TRIM25 antibody (Abcam, UK), and anti-GAPDH (Intechnology). After washed three times with TBST, the membranes were incubated with specified secondary antibody containing secondary antibodies labeled with HRP (BOSTER) at room temperature for 2 h. The band density was normalized to GAPDH and quantiﬁed by ImageJ software.

**T cell-mediated tumor cell killing assay**

To obtain activated T cells, peripheral blood mononuclear cells (PBMC) were cultured in RPMI-1640 medium and activated with anti-CD3 (CytoCares, China) and anti-CD28 (CytoCares, China) for one week according to the manufacturer’s protocol. The activated PBMCs were cultured with cancer cells at a ratio of 3:1. At 72 h after co-incubation, T cells and cell debris were removed by PBS, and living cancer cells were then quantified by crystal violet staining.

**Flow cytometry**

GC cells were seeded in 6-well plates. After reaching 70%-90% confluency, cells were transfected based on the manufacturer’s instructions of Lipofectamine 3000 reagents. After 72 h, cells were harvested and washed, cells were incubated with PE-labeled PD-L1 antibody (Biolegend, USA) for 30 min at 4°C in the dark. After washed 3 times, cells were analyzed by flow cytometry as soon as possible. The activated PBMCs were cultured with cancer cells and blocked with CD16/CD32 (Biolegend, USA) antibody. Cells were stained with CD45 (Biolegend, USA), CD3 (Biolegend, USA), CD4 (eBioscience, USA), CD8 (Biolegend, USA), IFN-γ (eBioscience, USA), TNF-α (eBioscience, USA), granzyme B (Biolegend, USA), PD-1 (eBioscience, USA), TIM-3 (eBioscience, USA) and LAG-3 (eBioscience, USA). Stained cells were analyzed by FACS Beckman CytoFLEX (CA, USA). All data were analyzed by FlowJo 10.0.

**Fluorescence in situ hybridization (FISH) and immunofluorescence**

For FISH assay, GC cells were fixed, blocked, hybridized. To detect co-localization of circRHBDD1 with indicated proteins, Cy-3-conjugated circRHBDD1 probes were incubated at 37°C in the dark overnight. Then, the cells were incubation with primary antibody at 4°C in the dark overnight. After washing three times, cells were incubation with secondary antibodies and DAPI for 30 min. The images were visualized by confocal microscope. The FISH probe sequence is shown as follows: circRHBDD1: 5’-AGGAAGTCCTCGGTCCCAGAGGCTG-3’.

For immunofluorescence assay, cells or tissues were washed three times with PBS and fixed with 4% paraformaldehyde, permeabilized with 0.5% Triton X-100, blocked in 3% bovine serum albumin, and incubated with the primary antibody 4°C in the dark overnight. After incubation with the secondary antibody, the images were collected using a fluorescence microscope (Carl Zeiss).

**RNA immunoprecipitation (RIP) and meRIP assay**

RIP assays were performed using a Magna RIP RNA-Binding Protein Immunoprecipitation Kit (Millipore) according to the manufacturer’s instructions. In brief, cells were lysed using RIP lysis buffer and incubated with antibodies against AGO2 (Abcam), IGF2BP2 (Abcam), or nonspeciﬁc IgG antibody (EMD Millipore), respectively, at 4°C overnight. According to the manufacturer's protocol, the meRIP analysis was carried out with Magna MeRIP m^6^A kit (EMD Millipore). The immunoprecipitated RNA was isolated and subjected to qRT-PCR analysis.

**RNA pull-down assay**

For RNA pull-down assay, the biotin-labeled circRHBDD1 probe was performed using a Magnetic RNA-protein Pull-down Kit (Thermo). The biotin-labeled sense and antisense probes were designed and synthesized by RiboBio. Briefly, total RNA was incubated with biotin-labeled probes for 5 min and streptavidin magnetic beads (Invitrogen) for 30 min with rotation. Unbound RNA was washed away, and RNA-protein binding buffer was added. The supernatant was obtained for mass spectrometry and Western blotting analysis.

**Animal models**

Three-to-four-week-old female C57BL/6 mice and nude mice were purchased from Nanjing Qinglong Mountain Experimental Animal Co. Ltd (Nanjing, China). All mice were kept in specific pathogen-free (SPF) facilities and all animal experiments approved by the Ethics Committee of Wannan Medical College. MKN-45 cells with circRHBDD1 knockdown and MKN-28 cells overexpressing circRHBDD1 in 100 μl (1 × 10^5^ cells) were subcutaneously implanted into the left flanks of nude mice. CircRHBDD1-silenced and circRHBDD1-overexpressing MFC cells in 100 μl (5 × 10^6^ cells) were subcutaneously implanted into the left flanks of C57BL/6 mice. Tumor length (L) and width (W) was measured every 3 days using a caliper and tumor volume was calculated as follows: V = L × W^2^/2. Eight weeks later, mice were sacriﬁced. Tumor samples were harvested and tumor weight and volume were recorded. Tumors tissues were analyzed by ﬂowcytometry analysis, FISH and immunofluorescence analysis.
