# Supplementary figures and images for: CircRHBDD1 promotes immune escape via IGF2BP2/PD-L1 signaling and acts as a nanotherapeutic target in gastric cancer
Source: J Transl Med. 2024 Jul 30;22:704. doi: 10.1186/s12967-024-05498-9 (PMC11289934; doi:10.1186/s12967-024-05498-9)

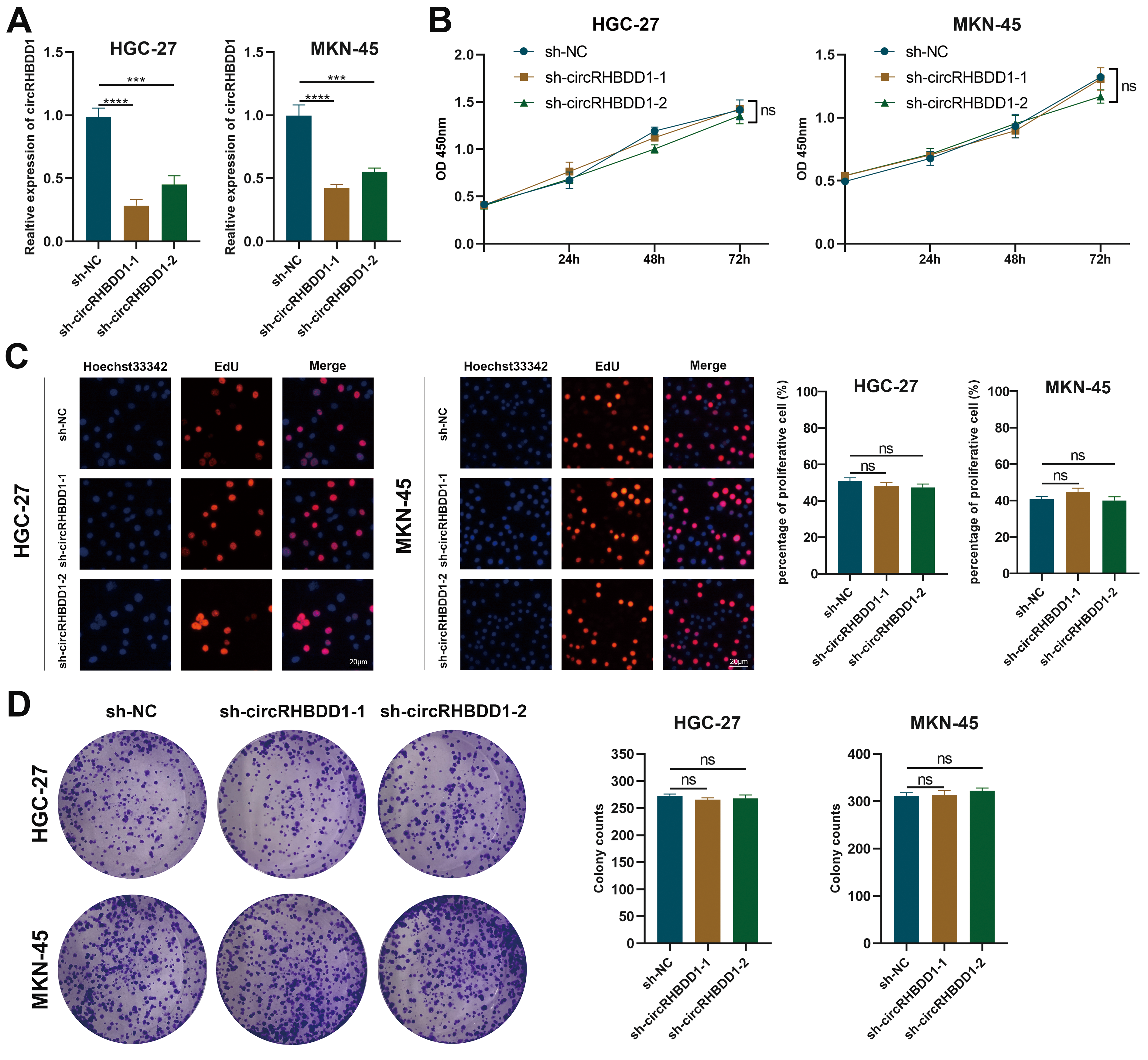

Supplement: Supplementary file 5 — Supplementary Material 5 [file 12967_2024_5498_MOESM5_ESM.tif]

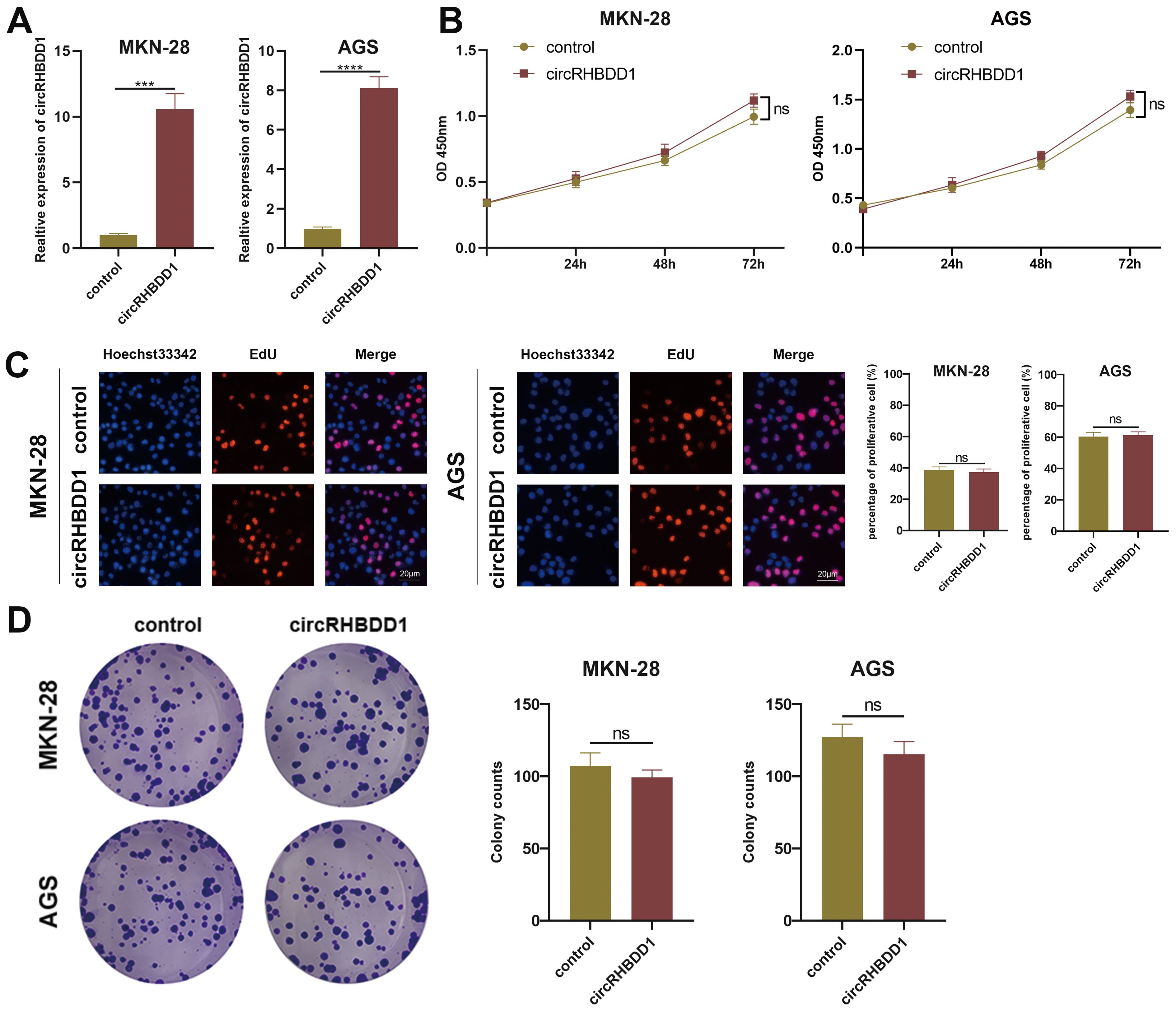

Supplement: Supplementary file 6 — Supplementary Material 6 [file 12967_2024_5498_MOESM6_ESM.tif]

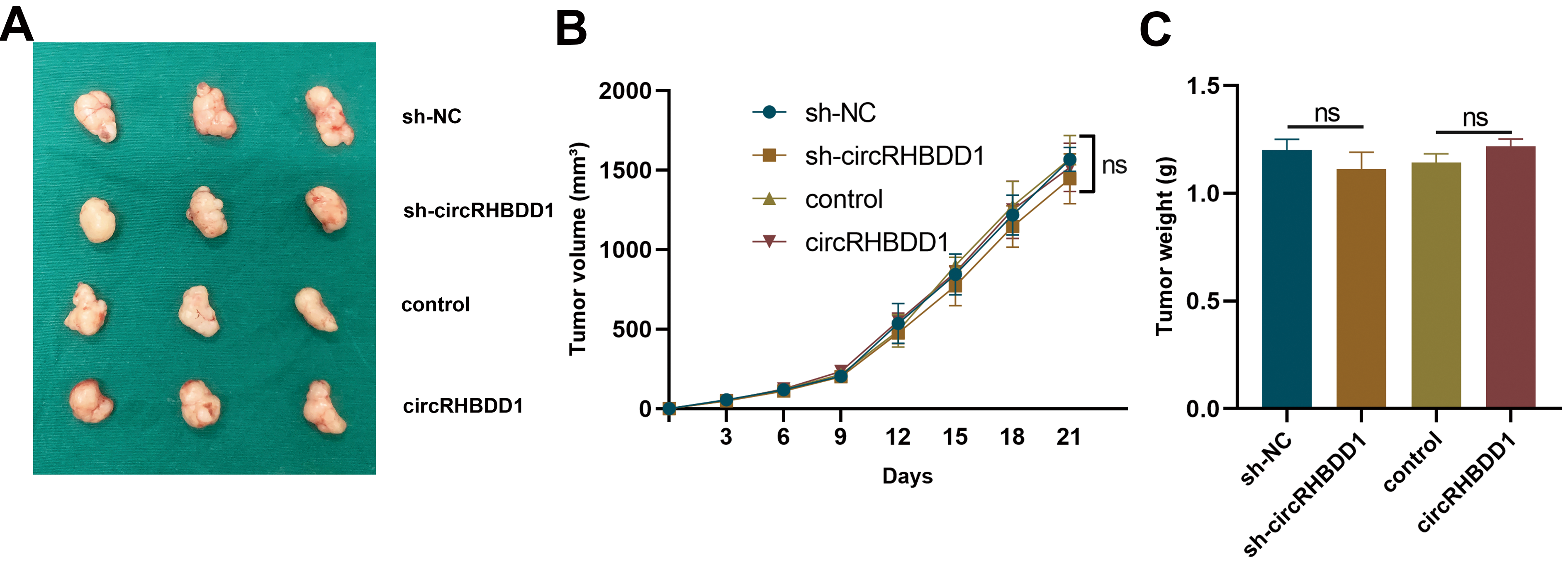

Supplement: Supplementary file 7 — Supplementary Material 7 [file 12967_2024_5498_MOESM7_ESM.tif]

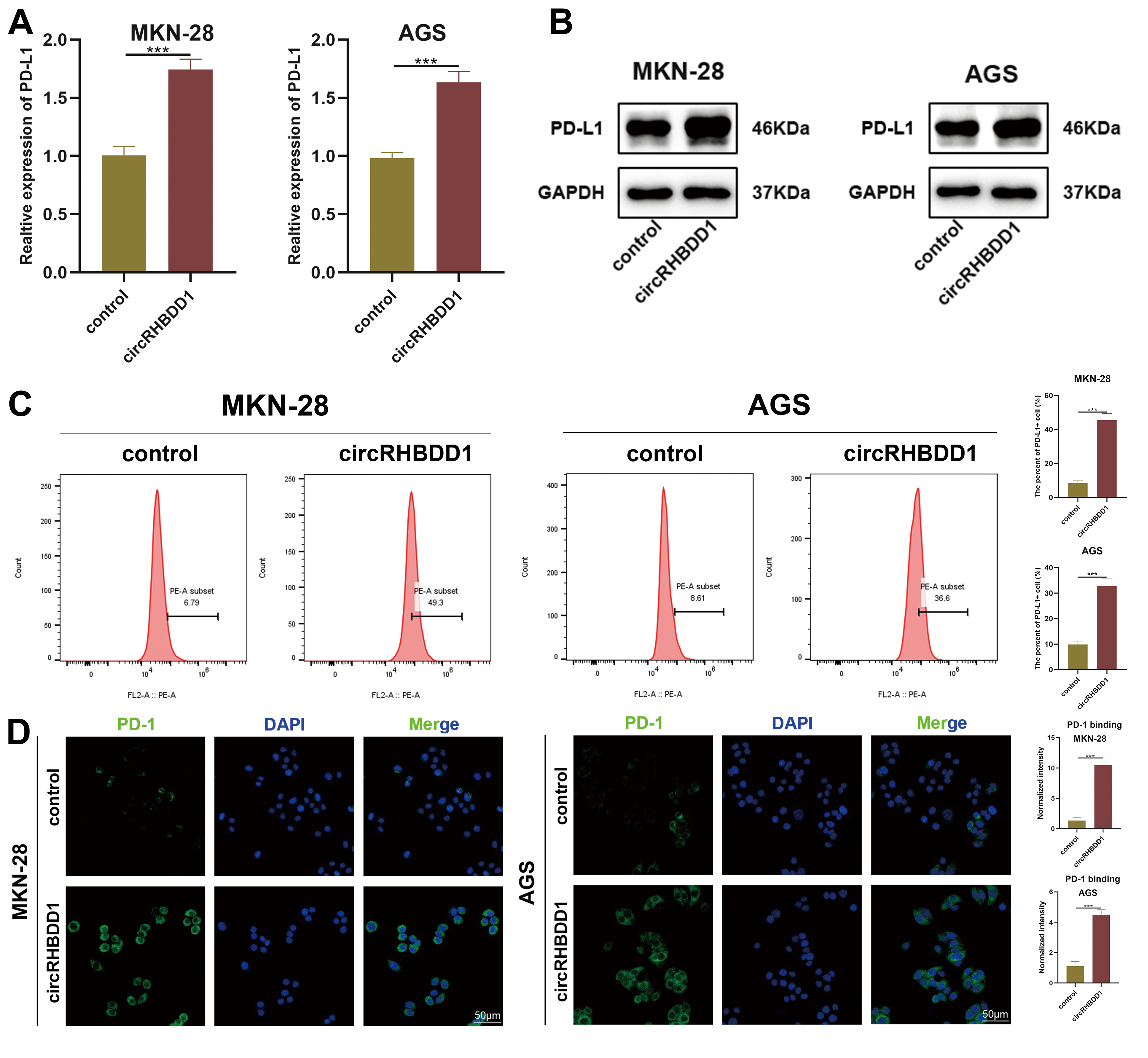

Supplement: Supplementary file 8 — Supplementary Material 8 [file 12967_2024_5498_MOESM8_ESM.tif]

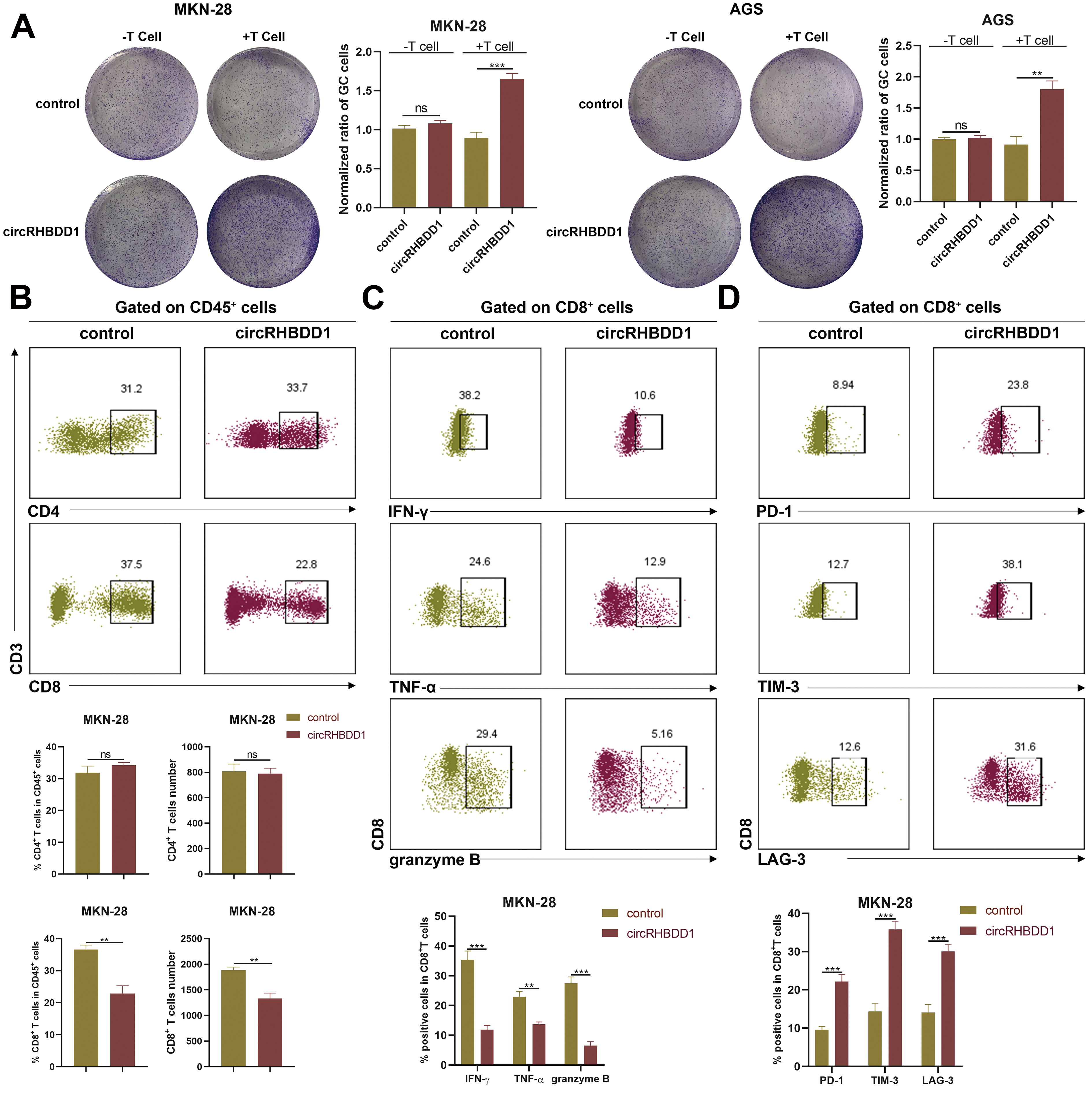

Supplement: Supplementary file 9 — Supplementary Material 9 [file 12967_2024_5498_MOESM9_ESM.tif]

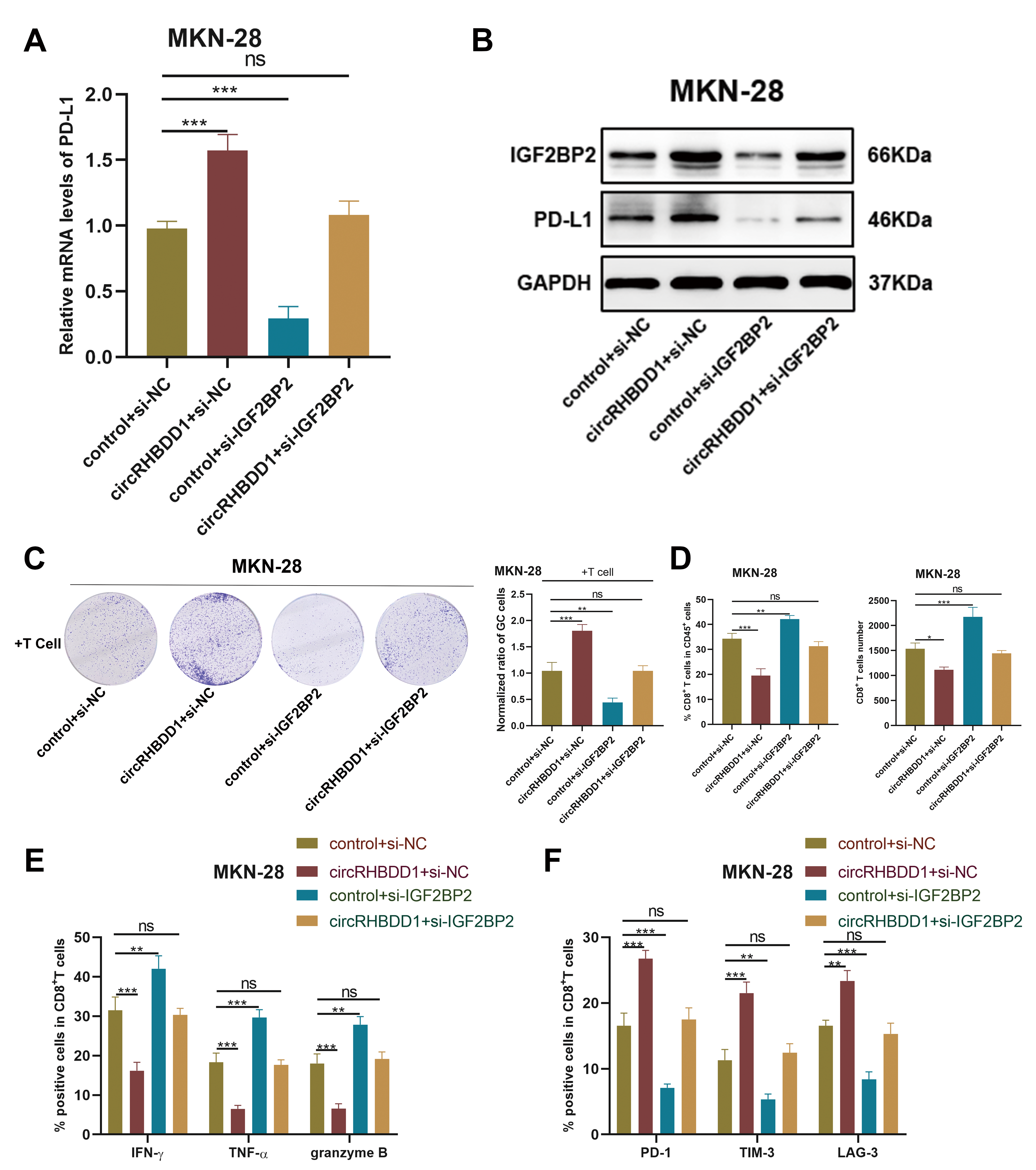

Supplement: Supplementary file 10 — Supplementary Material 10 [file 12967_2024_5498_MOESM10_ESM.tif]

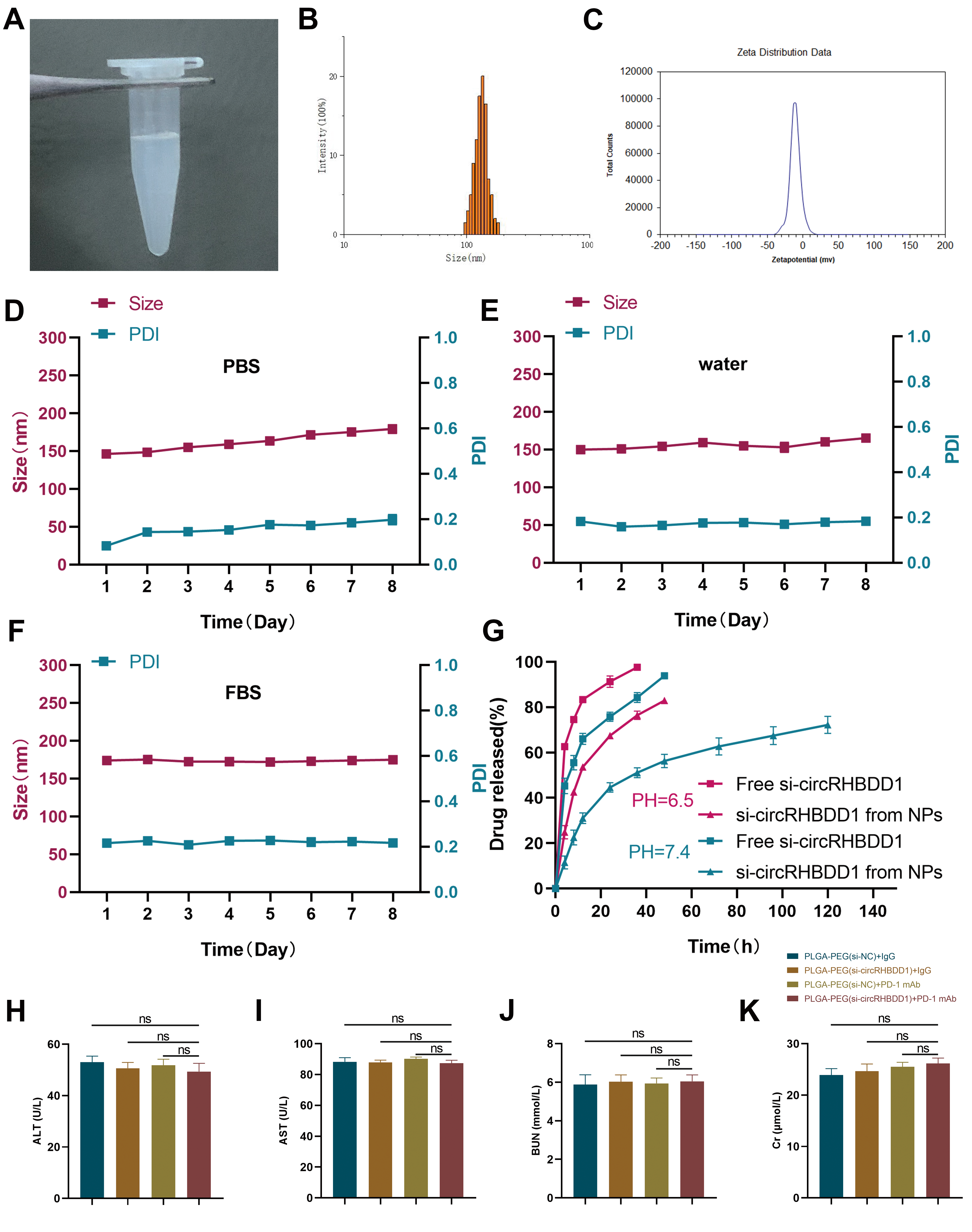

Supplement: Supplementary file 11 — Supplementary Material 11 [file 12967_2024_5498_MOESM11_ESM.tif]
